# Curcumin-encapsulated exosomes in bisphosphonate-modified hydrogel microspheres promote bone repair through macrophage polarization and DNA damage mitigation

**DOI:** 10.1016/j.mtbio.2025.101874

**Published:** 2025-05-15

**Authors:** Yunhui Si, Shuao Dong, Mengsha Li, Jiaying Gu, Manxuan Luo, Xiaohan Wang, Zhiwei Wang, Xiaorong Li, Chao Zhang

**Affiliations:** a School of Biomedical Engineering, Shenzhen Campus of Sun Yat-sen University, Shenzhen, Guangdong, 518107, PR China; b The Seventh Affiliated Hospital of Sun Yat-sen University, Shenzhen, Guangdong, 518107, PR China; c School of Materials Science and Engineering, Sun Yat-sen University, Guangzhou, Guangdong, 510275, PR China; d Southern Marine Science and Engineering Guangdong Laboratory (Zhuhai), Zhuhai, Guangdong, 519000, PR China

**Keywords:** MSC-Exos, Curcumin, Hydrogel, Osteogenesis, Immunomodulation

## Abstract

Repairing critical-sized bone defects represents a major challenge in clinical therapeutics due to inhibited osteogenic differentiation, harsh bone tissue microenvironment, and abnormal inflammatory response. Mesenchymal stem cell-derived exosomes (MSC-Exos) have demonstrated tremendous regenerative potential in tissue repair. However, the confined therapeutic efficacy, deficient targeting capability, and poor retention rate have rendered MSC-Exos-based cell-free therapies insufficient for clinical bone defect repair. This study prepared curcumin-loaded MSC-Exos (Cur@Exos) based on an endogenous drug delivery approach and encapsulated in bisphosphonate-modified GelMA hydrogel microspheres by microfluidics. The CE@BP-Gel microspheres demonstrated superior biocompatibility and were competent to accelerate biomineralization. The sustained-release Cur@Exos in the composite hydrogel microspheres actively regulated the polarization of RAW264.7 cells toward the regenerative M2 type and inhibited the osteoclastic activity, thereby creating an immune microenvironment suitable for osteogenesis. Meanwhile, the composite hydrogel microspheres can directly support the adhesion, proliferation and osteogenic differentiation of BMSCs and facilitate the migration and angiogenesis of HUVECs. *In vivo* experiments demonstrated that the CE@BP-Gel microspheres significantly accelerated the repair of critical-sized cranial bone defects in SD rats. The targets and mechanisms of action of CE@BP-Gel in bone immune regulation were investigated based on network pharmacology, molecular dynamics simulation and RNA sequencing. It was found that CE@BP-Gel mitigates DNA damage induced by ROS in inflammatory environments. The encapsulated curcumin enhances DNA damage repair by activating the TDP1 enzyme, consequently reducing the expression of inflammatory factors in macrophages. This study demonstrates a promising therapeutic strategy to design an exosome-based drug delivery system for bone defect repair.

## Highlights

• MSC-Exos with hydrogel microspheres boost curcumin delivery and bioavailability.
• CE@BP-Gel drives bone regeneration through immunomodulatory mechanisms.
• Curcumin activates the TDP1 enzyme to initiate DNA damage repair in macrophages.

## Graphical abstract

**Figure:**
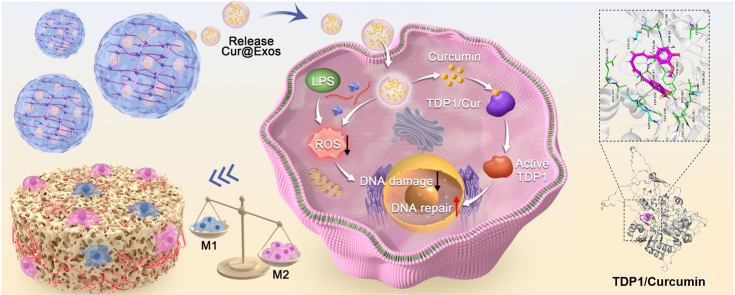

## 1 Introduction

Critical-sized bone defects represent a formidable clinical challenge characterized by extensive bone loss exceeding intrinsic healing capacity, typically resulting from high-energy trauma, oncologic resection, or septic complications [1,2]. These volumetric defects manifest as structural collapse, vascular insufficiency, and persistent inflammatory cascades that critically impair regeneration [[3], [4], [5]]. Dysregulated immune responses, marked by prolonged elevation of pro-inflammatory cytokines and excessive macrophage activation, create a hostile microenvironment that disrupts osteoblast-osteoclast equilibrium while promoting fibrous tissue formation [6,7]. Such pathological inflammation exacerbates cellular apoptosis, suppresses angiogenesis, and inhibits osteogenic differentiation of mesenchymal stromal cells, establishing a self-perpetuating barrier to bone repair [8,9]. Current clinical interventions, including autografts, allografts, and synthetic substitutes, often fail to address this immunopathological dimension; foreign body reactions to implanted materials amplify local inflammation and compromise graft integration [[10], [11], [12]]. Tissue engineering strategies face amplified challenges in critical-sized bone defects due to the dual requirements of modulating inflammatory signals and maintaining mechanical stability during extended healing periods [13]. Emerging evidence highlights the necessity of spatiotemporal immunomodulation, with scaffold systems incorporating anti-inflammatory agents or macrophage-polarizing cues demonstrating enhanced osteogenic outcomes through reprogramming of the defective immune microenvironment [[14], [15], [16]].

Seed cells represented by MSCs are widely accepted in bone tissue engineering for their multidirectional differentiation potential, immunomodulatory function, and ease of acquisition [17]. An increasing number of studies indicate that the therapeutic benefits of MSCs are primarily achieved through paracrine-produced exosomes [18,19]. MSC-Exos orchestrate angiogenesis and osteogenesis through the coordinated delivery of multifaceted cargoes encompassing regulatory proteins, signaling lipids, and epigenetic modulators [20,21]. Meanwhile, the smaller size of MSC-Exos can cross the extracellular matrix and tissue barriers to penetrate inflamed or damaged tissues. Compared with MSC therapy, MSC-Exos features lower tumorigenicity and immunogenicity, conferring higher safety in clinical applications [22]. In recent years, MSC-Exos has demonstrated significant application prospects in promoting bone tissue regeneration [23]. Y. Cui et al. revealed that MSC-Exos has an endogenous miRNA profile that promotes the expression of osteogenic genes, promotes the formation of H-type vessels, and inhibits osteoclast formation [24]. C. Sheng et al. found that osteogenic-induced adipose-derived MSC-Exos remarkably promoted the osteogenic differentiation of BMSCs via the miR-335-3p/Aplnr axis, reduced bone loss and improved bone microarchitecture in ovariectomy-induced osteoporotic mice [25]. Controlled preclinical studies have demonstrated that treatment with MSC-Exos increases bone mass, improves bone microstructure and enhances bone strength [26].

MSC-Exos-based cell-free therapies have progressed considerably in facilitating bone tissue regeneration, yet challenges remain in repairing critical-sized bone defects. Firstly, the therapeutic efficacy of naturally derived MSC-Exos is insufficient for critical-sized bone defect repair, especially in local microenvironments with severe imbalances in bone metabolism [27]. The osteogenic properties of MSC-Exos are affected by the cell source, culture conditions and extraction methods. Secondly, the unstable immunoregulatory capacity of MSC-Exos struggles to cope with critical-sized bone defects characterized by excessive ROS and abnormal levels of inflammation. The miRNA profile of MSC-Exos is significantly altered during the differentiation of MSC into osteoblasts or participation in bone repair due to the reprogramming of gene expression and the redistribution of organelles [28,29]. The alteration of miRNA profiles in MSC-Exos may result in unexpected immunomodulatory effects [30]. Finally, MSC-Exos are unable to specifically reach damaged bone tissue due to the deficiency of targeting capability and suitable release mechanisms. In repairing irregular bone defects, intravenous and in situ injections would cause a considerable elimination of exosomes by the immune system and lower local retention, resulting in limited therapeutic efficacy [31]. Hence, there is an urgent need to enhance the osteogenic activity and immunomodulatory properties of MSC-Exos and effectively deliver the optimized exosomes to the bone defect sites, synergistically modulating immunity and inducing vascularized bone regeneration.

Curcumin, a natural polyphenolic compound extracted from turmeric, has exhibited a number of biological activities, including antibacterial, anti-inflammatory, antioxidant and anti-tumor properties [32]. In recent years, the potential of curcumin in the treatment of orthopedic diseases has also attracted considerable attention [33,34]. It has been demonstrated that curcumin is capable of suppressing the generation of inflammatory factors, such as tumor necrosis factor-α (TNF-α), interleukin-1β (IL-1β), and interleukin-6 (IL-6), and these inflammatory factors play a crucial role in the pathogenesis of delayed bone repair [35]. In addition, the antioxidant properties of curcumin help alleviate oxidative stress, a major trigger of mitochondrial dysfunction in osteoblasts [36]. Curcumin can not only directly neutralize various types of free radicals but also further eliminate free radicals by increasing the activity of antioxidant enzymes *in vivo* [37]. On the other hand, curcumin has been reported to enhance osteoblast differentiation and activity while inhibiting osteoclast formation and activity, resulting in increased bone density and improved bone microarchitecture [38]. However, it is inescapable that the bioavailability of curcumin is greatly limited by its hydrophobic nature [39]. Poor solubility of curcumin at physiological pH levels leads to reduced absorption and rapid metabolism and clearance in the body [40]. Curcumin has shown insufficient therapeutic concentrations in preclinical and clinical trials at the target site, even when administered at fairly high doses [41]. Various strategies have been investigated to enhance the bioavailability of curcumin, including the application of nanotechnology, conjugation with carriers, and the development of its derivatives [[42], [43], [44]]. In fact, exosomes can serve as an excellent vehicle for curcumin loading and significantly enhance the bioavailability of curcumin. The lipid bilayer structure of exosomes can enhance the solubility of lipophilic curcumin [45]. Exosomes can further protect curcumin from rapid metabolism by enzymes *in vivo*, prolong its half-life in the blood circulation, and accordingly enhance its bioavailability. Furthermore, exosome-mediated membrane fusion or endocytosis pathways enable target cells to internalize curcumin-loaded exosomes, facilitating the direct intracellular delivery and subsequent functional activation of curcumin [46].

**Scheme 1:**
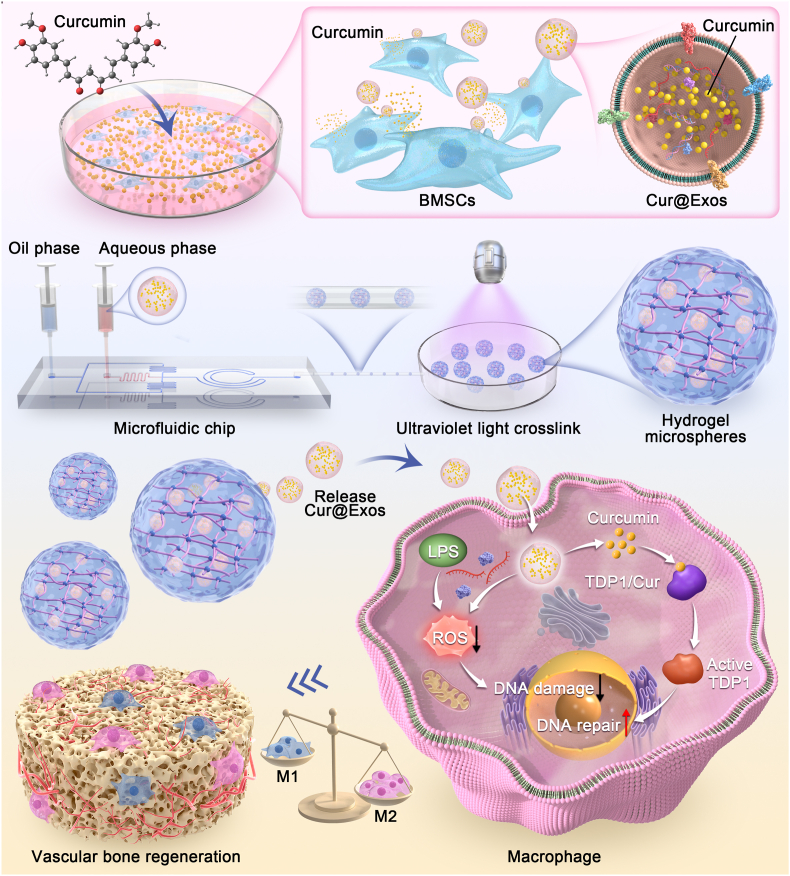
Schematic illustration of the preparation route and functional mechanism of CE@BP-Gel microspheres.

To address the clinical challenges in bone defect repair, we have developed a dual-functional delivery system (CE@BP-Gel) that integrates alendronate, a first-line clinical medication, with exosome-based bioactive factors. Specifically, Cur@Exos were synthesized via an endogenous drug-loading strategy and subsequently encapsulated within alendronate-modified GelMA hydrogel microspheres using microfluidic technology. The CE@BP-Gel microspheres exhibited enhanced biocompatibility and robustly supported the adhesion and proliferation of BMSCs. Mechanistically, CE@BP-Gel modulated the immune microenvironment by upregulating anti-inflammatory cytokines in macrophages and suppressing osteoclastogenesis while directly promoting BMSCs osteogenic differentiation and HUVECs angiogenesis, thereby facilitating vascularized bone regeneration. Further mechanistic studies revealed that the CE@BP-Gel system mitigated ROS accumulation and nuclear DNA damage in macrophages under inflammatory stimuli, with curcumin enhancing TDP1-mediated DNA repair efficiency and driving M2 macrophage polarization. The preparation route and functional mechanism of the CE@BP-Gel microspheres are illustrated in Scheme 1. The abovementioned findings collectively indicate that CE@BP-Gel has attractive application prospects in the repair of critical-sized bone defects.

## 2 Materials and methods

### 2.1 Materials

Type A gelatin (300 Bloom, reagent grade) was purchased from Sigma-Aldrich Trading Co., Ltd. (Shanghai, China). Methacrylic anhydride (C_8_H_10_O_3_, 94 %), Lithium Phenyl (2,4,6-trimethylbenzoyl) phosphinate (C_16_H_16_LiO_3_P, 98 %) and Alendronate sodium trihydrate (C_4_H_12_NaNO_7_P_2_·3H_2_O, 97 %) were purchased from Macklin Biochemical Technology Co., Ltd. (Shanghai, China). Span-80 and paraffin liquid were purchased from Aladdin Biochemical Technology Co., Ltd. (Shanghai, China). Curcumin (C_21_H_20_O_6_, 99 %) was purchased from MeilunBio Co., Ltd. (Shanghai, China). All chemicals were used as received without undergoing any additional purification processes. In the absence of specific guidelines, ultra-pure water was employed as the solvent throughout the experimental procedure.

### 2.2 Isolation of Cur@Exos

Cur@Exos were prepared via endogenous drug loading. As depicted in Fig. S1, based on the outcomes of cell proliferation activity and ALP activity, the optimal concentration of curcumin for incubation with BMSCs was determined to be 20 μM. BMSCs were incubated with 20 μM curcumin in a growth medium for 48 h. After washing with PBS to remove residual curcumin, cells were starved in a serum-free medium for 1 day. The conditioned medium was sequentially centrifuged at 300 g (10 min), 2000 g (20 min), and 10,000 g (30 min) at 4 °C to remove cellular debris. The supernatant was ultracentrifuged at 100,000 g for 1.5 h to pellet exosomes. Purified exosomes were resuspended in PBS, filtered through a 0.22 μm membrane, and ultracentrifuged again (100,000 g, 1.5 h). Cur@Exos were stored at −80 °C. Control exosomes (MSC-Exos) were isolated following identical procedures without curcumin treatment.

### 2.3 Fabrication of CE@BP-Gel microspheres

CE@BP-Gel microspheres encapsulating Cur@Exos were fabricated using a microfluidic emulsion method [47]. The aqueous phase consisted of 10 % (w/v) BP-GelMA in PBS containing 5 μg/μL exosomes and 0.5 % LAP (EFL, China). The oil phase comprised liquid paraffin with 5 % Span-80. Flow rates were set at 10 μL/min (aqueous phase) and 200 μL/min (oil phase). Droplets formed at the microfluidic chip outlet were crosslinked under 405 nm UV light. Microspheres were washed with 75 % ethanol and acetone to remove surfactants.

### 2.4 ALP activity and staining

BMSCs were seeded in 12-well plates at 5 × 10^4^ cells/well. After 24 h, the medium was replaced with OIM (α-MEM supplemented with 0.1 μM dexamethasone, 50 μM L-ascorbic acid, and 10 mM β-glycerophosphate sodium) containing hydrogel extracts. The medium was refreshed every 48 h. On day 7, cells were fixed with 4 % paraformaldehyde and stained using an ALP staining kit (Beyotime, China). For quantitative analysis, cells were lysed with RIPA buffer (Solarbio, China), and ALP activity was measured using a commercial kit (Yeasen, China), normalized to total protein content determined via BCA assay (Beyotime, China).

### 2.5 ARS staining

BMSCs were cultured in OIM with hydrogel extracts for 14 days to evaluate extracellular matrix (ECM) mineralization. Cells were fixed with 4 % paraformaldehyde for 30 min and stained with 0.1 % ARS solution (Solarbio, pH 4.2) for 30 min. After washing with PBS to remove excess dye, mineralized nodules were imaged using an inverted microscope.

### 2.6 Flow cytometry analysis of macrophage polarization

RAW264.7 cells were seeded in 6-well plates (1 × 10^5^ cells/well) and stimulated with 100 ng/mL lipopolysaccharide (LPS, Solarbio, China) for 24 h to induce an inflammatory phenotype. Cells were then treated with hydrogel extracts for 72 h. After harvesting, cells were incubated with FITC-conjugated anti-CD206 (BioLegend, USA) for 20 min in the dark, washed twice with 1 % BSA, and analyzed using a CytoFLEX flow cytometer (Beckman, USA). Data were processed using CytExpert software.

### 2.7 TRAP staining and activity assay

RAW264.7 cells were seeded in 6-well plates (1 × 10^5^ cells/well) and treated with 50 ng/mL RANKL (Novoprotein, China) and hydrogel extracts for 5 days to induce osteoclast differentiation. Cells were fixed with 4 % paraformaldehyde and stained using a TRAP staining kit (Solarbio, China). For quantitative TRAP activity, cells were lysed, and the supernatant was incubated with p-nitrophenyl phosphate substrate (Beyotime, China) at 37 °C for 1 h. Absorbance at 405 nm was measured to determine enzymatic activity.

### 2.8 Immunofluorescence staining

For macrophage polarization analysis, LPS-stimulated RAW264.7 cells were treated with hydrogel extracts for 72 h, fixed, permeabilized with 0.1 % Triton X-100, and blocked with 10 % goat serum. Cells were incubated overnight with anti-iNOS (1:150. Abcam, UK) or anti-CD206 (1:100, Abcam, UK), followed by Alexa Fluor 488-conjugated secondary antibodies. Cytoskeleton and nuclei were stained with FITC-phalloidin and DAPI, respectively. BMSCs cultured in OIM for 14 days were stained with anti-OCN (Abcam) for osteogenic markers. HUVECs were stained with anti-CD31 (Abcam).

### 2.9 *In vivo* bone regeneration assessment

Male Sprague-Dawley (SD) rats (8-week-old, weighing 200 g) were anesthetized with pentobarbital sodium (50 mg/kg) administered via intraperitoneal injection. Following standard aseptic preparation, where the surgical site was shaved and disinfected, a midline sagittal incision was performed along the scalp to expose the cranial region. A critical-sized circular full-thickness bone defect (5 mm diameter) was generated in the parietal bone using a trephine bur under continuous saline irrigation to minimize thermal damage. Particular care was taken to maintain periosteal integrity to preserve natural healing mechanisms. Postoperative closure was achieved through layered suturing, with subcutaneous administration of buprenorphine (0.05 mg/kg) for postoperative analgesia. The fabricated composite hydrogel microspheres were resuspended in sterile PBS at 20 mg/mL. During defect creation, 50 μL of hydrogel suspension (or equivalent volume of PBS for controls) was precisely injected into the defect cavity using a 26-gauge needle. Animals were randomly allocated into four experimental groups: Control (PBS), Gel, BP-Gel, and CE@BP-Gel (n = 3 per group). At 4 and 8 weeks post-implantation, euthanized specimens were collected and fixed in 4 % paraformaldehyde for 48 h prior to microarchitectural analysis using a high-resolution Micro-CT system (SkyScan 1272, Bruker; scanning parameters: 10 μm isotropic voxel size, 70 kV tube voltage).

Decalcification was performed in 10 % EDTA solution (pH 7.4) over 30 days with daily solution renewal for histological processing. Paraffin-embedded specimens were sectioned coronally at 5 μm thickness. Osteogenic differentiation was evaluated through immunostaining with primary antibodies against osteocalcin (OCN, 1:200 dilution, Abcam) followed by Alexa Fluor 488-conjugated secondary antibodies. Macrophage polarization analysis was performed using anti-iNOS antibodies (1:150 dilution, Proteintech) and anti-CD206 antibodies (1:100 dilution, Abcam). Nuclear counterstaining was achieved using DAPI, with fluorescence images acquired by CLSM. All experimental protocols complied with institutional guidelines and were approved by the Animal Ethics Committee of Shenzhen Institute for Drug Control (Protocol No. 20240718).

### 2.10 Network pharmacology and molecular docking

Retrieve the Isomeric SMILES value of curcumin from the PubChem database and download its 3D structure in sdf format. Next, import the Isomeric SMILES into the SwissTargetPrediction and SuperPred databases for target prediction. Additionally, input the compound name into the Comparative Toxicogenomics Database (CTD) to predict potential targets. Unify the predicted targets and import them into UniProt for conversion into standardized gene names. After removing duplicates, compile a list of curcumin's targets. Perform keyword searches for “osteogenesis”, “osteogenic differentiation”, “bone regeneration” and “osteoporosis” in the GeneCards and OMIM databases to identify osteoporosis-related targets. Summarize and remove duplicates to obtain a comprehensive list of osteoporosis-associated targets. Identify the intersection between curcumin's targets and osteoporosis-related targets. Import these intersecting targets into the STRING database, select “Multiple proteins” and specify “Human sapiens” as the species to generate a protein-protein interaction (PPI) network. Import the resulting TSV file into Cytoscape 3.10.2 for further analysis of the PPI network and use the CentiScaPe 2.2 plugin to screen for core targets based on Degree, Betweenness, and Closeness centrality measures. Import the core targets into the DAVID database, specifying “Human sapiens” as the species, to perform the GO function and KEGG pathway enrichment analysis. Select significant enrichment results with P < 0.01 and visualize these results using the MicroBioMe online platform. Analyze the GO and KEGG pathway enrichment diagrams. Finally, the potential curcumin targets related to osteoporosis are imported into Cytoscape 3.10.2 to construct a drug-intersection target-pathway-disease network relationship diagram.

Molecular docking provides robust evidence for the interaction between small molecules and proteins. To validate curcumin as a potential promoter of immunoregulation, we evaluated its docking accuracy with the candidate target protein TDP1. The structure of TDP1 was retrieved from the Protein Data Bank (PDB), and all water molecules and non-essential ligands were removed using PyMOL. Subsequently, the three-dimensional structure of curcumin was docked with TDP1 using AutoDock Tools, and the resulting binding modes were visualized and analyzed using PyMOL.

### 2.11 Statistical analysis

Data are expressed as mean ± SD (n ≥ 3). Statistical significance was determined via one-way ANOVA with Tukey's test (GraphPad Prism 8.0). All research data were checked with respect to normal distribution. Significance levels: ∗p < 0.05, ∗∗p < 0.01, ∗∗∗p < 0.001, ∗∗∗∗p < 0.0001, and ns: no significant.

## 3 Results and discussion

### 3.1 Preparation and characterization of CE@BP-Gel microspheres

**Fig. 1:**
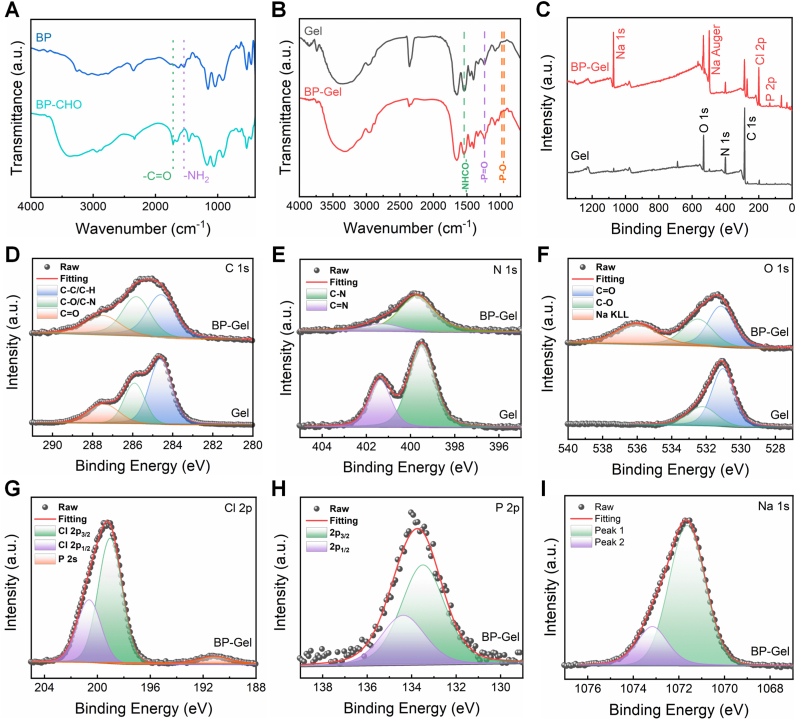
Characterization of bisphosphonate-modified GelMA hydrogels. (A) FTIR spectra of the aldehyde-modified BP. (B) FTIR spectra of the BP-Gel. (C) XPS survey spectra of the BP-Gel. (D–I) High-resolution XPS spectra of (D) C 1 s, (E) N 1s, (F) O 1s, (G) Cl 2p, (H) P 2p and (I) Na 1s for the BP-Gel.

Bisphosphonates (BP), a class of drugs extensively utilized in clinical practice for treating osteoporosis and other bone-related diseases, suppress osteoclast activity and indirectly enhance bone formation, thereby mitigating bone loss, increasing bone density, and reducing fracture risks [48]. Currently, common bisphosphonate drugs include alendronate, risedronate, ibandronate, and zoledronic acid [49]. In this work, sodium alendronate was grafted onto GelMA molecules, and the hydrogel microspheres could be injected in situ into the defect area to achieve sustained local release of BP. Referring to the previous report by Z. Zhao et al., BP was reacted with an excess of glutaraldehyde to obtain aldehyde-modified BP (BP-CHO) [47]. Subsequently, the aldehyde groups of BP-CHO were conjugated with the amino groups of GelMA via Schiff base reaction to form GelMA-BP. As illustrated in Fig. 1(A), after aldehyde modification, the N-H in-plane bending vibration (1550 cm^−1^) attributed to the amino group in BP significantly decreased, while a distinct carbonyl (C=O) stretching vibration at 1724 cm^−1^ was observed. The reduction of the amino group and the appearance of the carbonyl group indicate that the aldehyde group was successfully grafted onto BP via the amino group, confirming the successful preparation of BP-CHO.

Fig. 1(B) presents the infrared spectrum of BP-Gel, where the P=O peak at 1234 cm^−1^ and the P-O peak at 972 cm^−1^ were significantly enhanced, while the intensity of the amide II band (N-H bending vibration and C-N stretching vibration) near 1550 cm^−1^ slightly decreased. The enhanced signals of the phosphate groups in BP-Gel indicate the successful grafting of BP onto GelMA molecules. Furthermore, XPS analysis was performed to further characterize the bisphosphonate-modified GelMA (BP-Gel). As shown in Fig. 1(C), the XPS survey spectra of BP-Gel revealed the presence of P and Na elements derived from sodium alendronate, which were almost absent in Gel. In the high-resolution XPS spectra, the sub-peaks of C 1s and N 1s in BP-Gel were consistent with those in Gel, with only slight differences in relative proportions (Fig. 1(D, E)). Notably, the Auger peak of Na was observed in the O 1s spectrum of BP-Gel but not in that of Gel, further confirming the presence of sodium salt in BP-Gel (Fig. 1(F)). Additionally, the presence of P 2s was observed in the Cl 2p XPS spectrum of BP-Gel, highlighting the abundance of P elements (Fig. 1(G)). Moreover, strong P 2p and Na 1s signals were detected in BP-Gel but were hardly identifiable in Gel (Fig. 1(H, I)). These results collectively demonstrate the successful grafting of BP onto GelMA molecules.

The molecular weight of GelMA is dependent on both the molecular weight of its precursor gelatin and the degree of amino group substitution. For GelMA with 90 % amino substitution, the molecular weight typically ranges between 100 and 200 kDa. While the methacryloylation modification introduces additional functional groups, the overall molecular weight remains predominantly influenced by the original gelatin chain length. Gel strength, as characterized by the Bloom index measured via standardized Bloom gelometer testing, reflects the capacity of gelatin to maintain structural stability in its gelled state. This index demonstrates a positive correlation with average molecular weight. Commercial gelatin generally exhibits Bloom values ranging from 90 to 300 g, with the porcine skin-derived type A gelatin employed in this study demonstrating superior strength through its 300 g Bloom value. Optimized mechanical properties of GelMA hydrogels are achieved through controlled methacryloylation degree and precursor concentration. The crosslinking density of GelMA hydrogel increases proportionally with both the degree of substitution (DS) and pre-polymer concentration, though this enhancement occurs at the expense of injectability [50]. Following established protocols, this study utilized GelMA at 10 % (w/v) concentration with approximately 90 % amino substitution [51,52]. The resultant GelMA hydrogel microspheres exhibit exceptional injectability, favorable mechanical integrity, and an interconnected porous architecture. These biomimetic properties effectively mimic critical aspects of the extracellular matrix, thereby promoting BMSCs adhesion, proliferation, and differentiation, which are essential processes for supporting bone tissue regeneration.

**Fig. 2:**
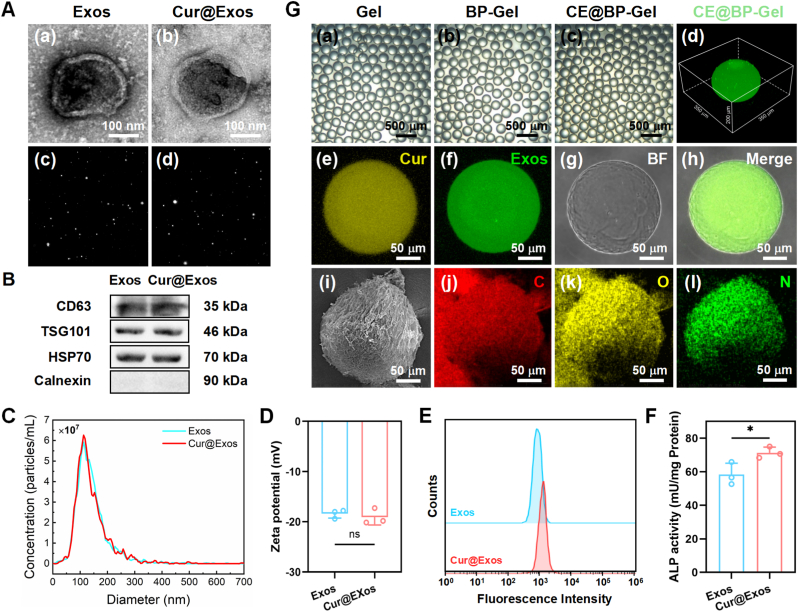
Characterization of Cur@Exos and CE@BP-Gel microspheres. (A) TEM and Brownian motion images of the Exos (a, c) and Cur@Exos (b, d). (B) Western blot identification of the surface marker proteins of exosomes. (C) Nanoparticle tracking analysis of the concentration and particle size distribution of the exosomes. (D) Zeta potentials of Exos and Cur@Exos. (E) Immunomodulatory properties of Cur@Exos based on flow cytometry analysis. (F) ALP activity of BMSCs induced by Cur@Exos. (G) Characterization of the hydrogel microspheres, (a–c) optical images of the hydrogel microspheres, (d) three-dimensional profile of the hydrogel microsphere encapsulating PKH-67 labeled Cur@Exos, (e–h) confocal laser microscope observations of the fluorescently labeled CE@BP-Gel microspheres, (i–l) element distribution images of the CE@BP-Gel microspheres. n = 3, ∗p < 0.05 and ∗∗p < 0.01.

Exosomes are extracellular vesicles with diameters ranging from 30 to 150 nm, released through the fusion of multivesicular bodies with the cell membrane [53]. As shown in Fig. 2(A), both MSC-Exos (Exos) and curcumin-loaded exosomes (Cur@Exos) exhibit a spherical vesicle structure with a clear bilayer membrane, and their particle size is approximately 150 nm. The endogenous curcumin-loading method did not disrupt the morphology or structure of the exosomes, thereby preserving the excellent biocompatibility and low immunogenicity of MSC-Exos. As illustrated in Fig. 2(B), western blot analysis confirmed the expression of exosomal surface marker proteins. Exos and Cur@Exos showed significant CD63, TSG101, and HSP70 expression, while the endoplasmic reticulum-specific membrane protein Calnexin was not detected. This result indicates the high purity of the extracted exosomes, free from contamination by cell lysates. NTA results revealed that the size distribution of the two exosomes ranged from 50 to 250 nm, with an average diameter of around 150 nm (Fig. 2(C)). Additionally, there was no significant difference in the concentration of the two exosomes. As shown in Fig. 2(D), the determined zeta potentials were −18.3 mV for MSC-Exos and −19.1 mV for Cur@Exos. The comparable absolute surface potential values before and after curcumin loading indicate preserved colloidal stability following therapeutic agent incorporation. The hydrophobic nature of curcumin facilitates its predominant localization within the exosomal lumen, thereby exerting minimal influence on surface charge characteristics.

Due to the notable anti-inflammatory properties of curcumin, Cur@Exos exhibited enhanced immunomodulatory performance compared to Exos. As presented in Fig. 2(E), Cur@Exos promoted the secretion of CD206 in LPS-induced RAW264.7 cells. The increased CD206 expression facilitates the transition of macrophages from a pro-inflammatory to an anti-inflammatory phenotype, accelerating inflammation resolution and tissue repair [54]. Furthermore, Cur@Exos demonstrated enhanced osteogenic activity, as evidenced by significantly increased alkaline phosphatase activity in BMSCs cultured with Cur@Exos (Fig. 2(F)). Following the successful extraction of Cur@Exos, they were encapsulated into BP-modified GelMA hydrogel microspheres. As illustrated in Fig. 2(G), Gel, BP-Gel, and CE@BP-Gel exhibited spherical particle morphology with similar particle sizes. The inset (d) illustrates the three-dimensional distribution of Cur@Exos within the hydrogel microspheres. PKH-67-labeled Cur@Exos (green fluorescence) were uniformly distributed within the hydrogel microspheres, allowing for clear visualization of the microsphere contours. Due to the intrinsic fluorescence of curcumin, the loading of curcumin (yellow) and exosomes (green) within the hydrogel microspheres could be observed via fluorescence imaging, as shown in the insets of (e-h). These results further confirm the successful preparation of CE@BP-Gel microspheres. Elemental mapping of CE@BP-Gel revealed a uniform distribution of C, O, and N elements (insets i-l in Fig. 2(G)). However, the presence of P element was not identified due to the detection limits of energy-dispersive X-ray spectroscopy. Semi-quantitative XPS analysis indicated that the atomic percentages of P in Gel and BP-Gel were 0.15 % and 3.15 %, respectively. The high-resolution SEM imaging of CE@BP-Gel was illustrated in Fig. S2. The CE@BP-Gel microspheres exhibited a porous architecture with surface pore dimensions ranging from 10 to 20 μm. The three-dimensional porous network facilitates efficient diffusion of oxygen, nutrients, and metabolic waste while providing structural support for cellular proliferation. This interconnected framework creates a biomimetic microenvironment conducive to cellular activities at bone defect sites, particularly favoring the migration and adhesion of primary cells or stem cells through its topography-mediated biological interactions.

**Fig. 3:**
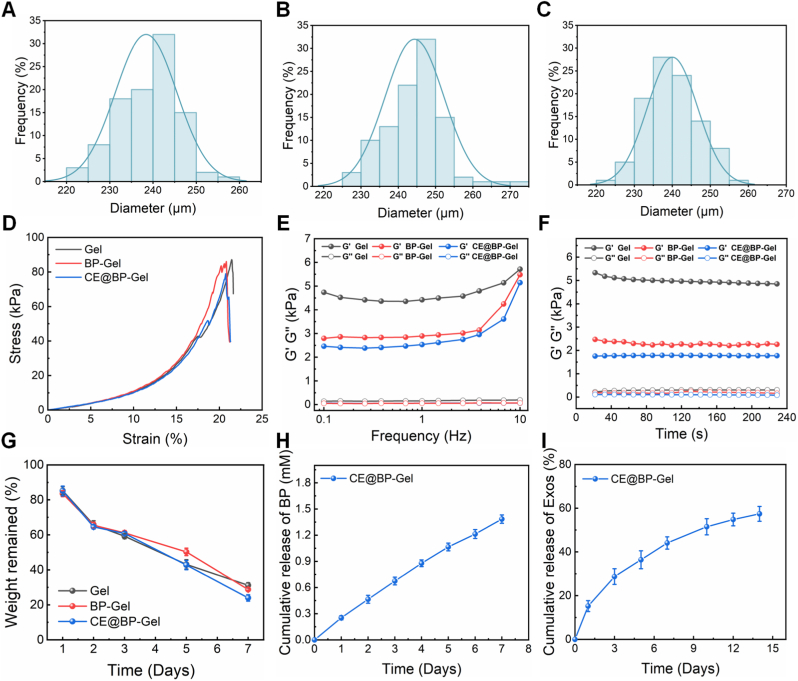
Characterization of the hydrogel microspheres. Particle size distribution of (A) Gel, (B) BP-Gel and (C) CE@BP-Gel. (D) Compressive stress-strain curves of the hydrogels. (E) Rheological profile over a frequency scan. (F) Rheological profile over a time scan. (G) Degradation behavior of hydrogel microspheres in PBS containing 0.5 U/mL type II collagenase and 100 mM H_2_O_2_. (H) Release profile of BP in the CE@BP-Gel microspheres. (I) Release profile of exosome in the CE@BP-Gel microspheres.

The particle size of hydrogel microspheres determines the surface area-to-volume ratio, thereby influencing their mechanical and responsive properties. As presented in Fig. 3(A–C), the average particle sizes of Gel, BP-Gel, and CE@BP-Gel microspheres were 238.5 μm, 242.1 μm, and 240.3 μm, respectively. The modification of BP and the loading of Cur@Exos did not significantly affect the particle size of GelMA hydrogel microspheres. Consequently, the differences in biological properties among different hydrogel microspheres primarily stemmed from the differences in components rather than particle size. As illustrated in Fig. S3, the CE@BP-Gel composite demonstrates excellent injectability, enabling minimally invasive delivery of hydrogel microspheres to bone defect sites. This characteristic proves particularly advantageous for bone tissue repair. To assess the impacts of BP modification and the introduction of Cur@Exos on the mechanical properties of GelMA hydrogels, cylindrical bulk hydrogel specimens were fabricated for compression and rheological performance tests. The compression test in Fig. 3(D) demonstrated that the mechanical properties of the three hydrogels were comparable, with an average compressive modulus of 23.5 kPa. In the present study, the compressive modulus of CE@BP-Gel was conspicuously lower than the mechanical strength of cancellous bone or cortical bone. This characteristic suggests that the current design is more suitable for non-load-bearing bone defect repair (e.g., cranial reconstruction or intraosseous filling). Nevertheless, CE@BP-Gel exhibits degradation kinetics synchronized with cellular migration and extracellular matrix deposition cycles (approximately 2–4 weeks) during early-stage bone regeneration. Its moderate degradation rate maintains temporary mechanical support for nascent bone tissue while preventing premature structural collapse [55]. Furthermore, the bisphosphonate component within CE@BP-Gel expedites mineralization and facilitates the progressive reinforcement of mechanical strength (Fig. S4). Mechanical strength was elevated after 14 days of in vitro mineralization, with the compressive modulus increasing to 28.3 kPa.

The rheological performance tests in Fig. 3(E, F) indicated that the storage modulus (G′) of the three hydrogels was far greater than the loss modulus (G″). This implies that when the hydrogels were subjected to periodic stress, their elastic response exceeded the viscous response, thereby enabling better restoration to the original shape after mechanical deformation [56]. The grafting of BP onto GelMA molecules influenced the crosslinking density of the hydrogels, thus resulting in a decrease in the storage modulus of BP-Gel and CE@BP-Gel compared to Gel but remaining much higher than their loss modulus. Meanwhile, the encapsulation of exosomes had a relatively minor influence on the rheological properties of the hydrogels. In the frequency sweep mode, from low to high frequencies, the G′ of the three hydrogels rose, while the G″ remained unchanged, signifying that the prepared hydrogels exhibited high rigidity. After implantation *in vivo*, the higher rigidity of the hydrogel microspheres could maintain their shape and structure when subjected to periodic loading without dissipating excessive energy due to viscous flow. Additionally, in the time sweep mode over 240 s, the G′ and G″ of the three hydrogels underwent no significant changes, suggesting that the prepared hydrogels all possessed good mechanical stability.

The biodegradation performance of the hydrogel microspheres was assessed in PBS containing collagenase and H_2_O_2_, as presented in Fig. 3(G). All three hydrogels were witnessed to undergo slow degradation in PBS at 37 °C, and the mass loss was approximately 3 % after 14 days (Fig. S5). To simulate ROS generation during acute inflammatory responses post-implantation, degradation tests were conducted in PBS supplemented with 100 mM H_2_O_2_, revealing accelerated degradation kinetics with 37 % mass loss by day 14. For long-term degradation behavior evaluation, type II collagenase was introduced into PBS to mimic enzymatic degradation *in vivo*. While physiological collagenase activity in healthy tissues remains low (0.01–0.1 U/mL), transient elevation up to 0.1–0.2 U/mL may occur in bone defect or inflammatory microenvironments. In alignment with established protocols for GelMA hydrogel degradation studies, a collagenase concentration of 0.5 U/mL was selected to accelerate degradation while maintaining biological relevance [[57], [58], [59]]. CE@BP-Gel exhibited 50 % mass loss after 7 days in 0.5 U/mL collagenase-containing PBS, confirming favorable degradability. Synergistic degradation effects were observed when combining oxidative and enzymatic challenges. Co-incubation with H_2_O_2_ and collagenase induced 77 % mass loss within 7 days, demonstrating that elevated ROS levels and collagenase activity cooperatively enhance hydrogel degradation. The favorable biodegradability of the hydrogel microspheres can ensure the gradual release of internal drugs or growth factors, thereby facilitating the repair of damaged bone tissue.

Meanwhile, the pH value during the degradation process of the hydrogel microspheres was continuously monitored (Fig. S6). The CE@BP-Gel system maintained a stable pH of 7.2 after 14-day degradation in PBS, indicating that the slow degradation rate of GelMA hydrogel microspheres in the physiological buffer does not significantly alter solution acidity. To better simulate inflammatory conditions and enzymatic degradation in bone defect microenvironments, parallel experiments were conducted in PBS supplemented with 100 mM H_2_O_2_ and 0.5 U/mL type II collagenase, with continuous pH recording. The enzymatic activity of type II collagenase specifically cleaves the Pro-X-Gly-Pro sequences (where X represents neutral amino acids) in gelatin, generating short peptide fragments. The inherent abundance of weakly acidic amino acids (e.g., glutamic acid and aspartic acid) in gelatin facilitates pH reduction through carboxyl group liberation during degradation [60]. Furthermore, the methacrylate ester bonds in GelMA undergo hydrolytic cleavage under H_2_O_2_-mediated oxidative conditions, producing carboxylic acid derivatives such as methacrylic acid that contribute to system acidification. Consequently, a progressive pH decrease to 6.4 was observed in CE@BP-Gel samples degraded for 14 days in PBS containing both H_2_O_2_ and collagenase II. Notably, the amino acids and oligopeptides generated from *in vivo* GelMA hydrogel degradation demonstrate favorable biocompatibility, as biological systems can effectively metabolize and assimilate them without long-term accumulation [61]. Furthermore, the release behavior of the active components in CE@BP-Gel in a simulated physiological environment was evaluated. As presented in Fig. 3(H), BP exhibited an almost linear cumulative release within one week, with a nearly constant release rate. The constant release of BP can prevent unstable therapeutic effects or side effects caused by fluctuations in drug concentration. Meanwhile, Cur@Exos in CE@BP-Gel achieved a sustained release over two weeks (Fig. 3(I)). The three-dimensional network structure of the hydrogel is conducive to the slow release of exosomes and maintains their biological activity. The CE@BP-Gel microspheres can protect exosomes from enzyme degradation *in vivo*, extend the half-life, and thereby continuously release at the defect site to promote bone regeneration.

The swelling behavior of composite hydrogels in PBS was also investigated, as illustrated in Fig. S7. All three hydrogels reached swelling equilibrium within 24 h, demonstrating equilibrium swelling ratios of 269 %, 275 %, and 236 % for Gel, BP-Gel, and CE@BP-Gel, respectively. The surface components of exosomes, including proteins and lipids, may establish supplementary crosslinking sites through non-covalent interactions (hydrogen bonding and hydrophobic interactions) with GelMA polymer chains. This interfacial interaction enhances hydrogel network compactness by restricting water molecule permeation and network expansion, thereby explaining the reduced swelling ratio observed in CE@BP-Gel. In bone defect repair applications, excessive swelling ratios may lead to hydrogel overexpansion and subsequent displacement, whereas the reduced swelling capacity of CE@BP-Gel mitigates these risks while maintaining precise defect filling. Before clinical implantation, hydrogel microspheres were pre-equilibrated in PBS for 24 h to achieve dimensional stability through complete swelling equilibrium.

### 3.2 Biocompatibility assessment of hydrogel microspheres

**Fig. 4:**
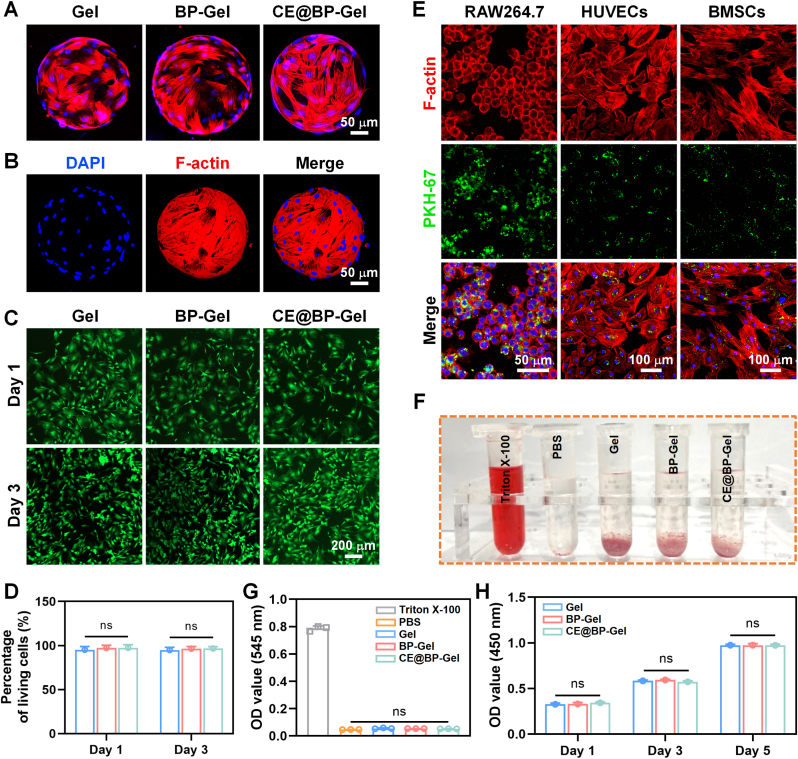
Biocompatibility evaluation of the hydrogel microspheres. (A) Morphological observation of BMSCs on the hydrogel microspheres after 24 h of incubation. (B) Three-dimensional profile of BMSCs adhered to the CE@BP-Gel surface. (C) Live/dead staining of BMSCs incubated with the hydrogel microspheres. Live cells were green, and dead cells were red. (D) Quantitative statistics of the percentage for living cells. (E) Uptake of exosomes in RAW264.7, HUVECs, and BMSCs incubated with CE@BP-Gel. (F) Photograph of the hemolysis experiment. (G) Hemolysis test of red blood cells incubated with the hydrogel microspheres. (H) Proliferation of BMSCs incubated with the hydrogel microsphere extracts. (For interpretation of the references to colour in this figure legend, the reader is referred to the Web version of this article.)

Excellent biocompatibility constitutes an essential prerequisite for the clinical application of biomaterials. Biocompatibility implies that the implanted material should not induce toxicity, inflammation, allergy, or immune rejection. As illustrated in Fig. 4, the cytocompatibility of the fabricated composite hydrogel microspheres was meticulously evaluated. As depicted in Fig. 4(A and B), following 24 h of incubation, BMSCs fully spread on the surface of the hydrogel microspheres, and the cells were interconnected via filopodia. There was no significant difference in the number of adherent cells among the groups, signifying the excellent biocompatibility of the prepared hydrogel microspheres. Concurrently, the live/dead staining assessment indicated that after incubation with the hydrogel microsphere extract for 1 and 3 days, the survival rate of BMSCs in each group was approximately 100 %, demonstrating that the fabricated hydrogel microspheres possessed almost no cytotoxicity (Fig. 4(C, D)).

Confocal imaging was employed to observe the uptake of exosomes by cells after incubation with the hydrogel microspheres, as shown in Fig. 4(E). Efficient uptake of exosomes was detected in all three types of cells closely associated with bone repair. Compared with BMSCs and HUVECs, a larger number of exosomes were taken up by RAW264.7 cells. The difference in the amount of exosomes uptake primarily originated from the variances in the uptake mechanisms of the cells. Macrophages are dedicated immune system phagocytes and actively uptake exosomes through receptor-mediated endocytosis and micropinocytosis [62]. BMSCs mainly rely on clathrin-dependent endocytosis or micropinocytosis, with a lower uptake efficiency than the active phagocytic mechanism [63]. HUVECs mainly uptake exosomes through caveolin-mediated endocytosis, and the distribution density of membrane surface receptors restricts the efficiency [64]. Hence, based on the efficient uptake of exosomes by macrophages, targeted modulation of the active components in exosomes is anticipated to achieve immune regulation-mediated bone regeneration and angiogenesis.

Hemocompatibility is essential to prevent thrombus formation, hemolysis, or coagulation triggered by the implanted material. As presented in Fig. 4(F), the hydrogel microspheres were co-incubated with red blood cell suspensions, and the content of hemoglobin released upon red blood cell rupture was detected. The Triton X-100 and PBS groups were set as positive and negative control groups, respectively. Quantitative tests based on hemoglobin absorbance demonstrated that the hemolysis rate of all hydrogel microspheres was less than 5 %, indicating excellent blood compatibility (Fig. 4(G)). Additionally, the proliferation activity of BMSCs incubated with different hydrogel microspheres was quantitatively assessed based on the CCK-8 assay and presented in Fig. 4(H). BMSCs in all groups exhibited favorable proliferation activity at the 1, 3, and 5 days, and there was no significant difference among the groups.

Regarding material composition, GelMA is enzymatically degraded into amino acids and oligopeptides, natural gelatin derivatives with well-documented biocompatibility and negligible toxicity risks [65,66]. Alendronate sodium, chemically conjugated to GelMA via Schiff base formation, may release free bisphosphonate molecules during degradation. The localized delivery system provided by hydrogel microspheres enables sustained alendronate release at implantation sites, effectively reducing systemic exposure risks while maintaining therapeutic efficacy with no observable toxicity in experimental models [67]. MSC-Exos exhibit low immunogenicity, with their lipid and protein degradation products being metabolized through physiological pathways [68]. Curcumin encapsulation within exosomes enhances its bioavailability, with primary metabolites identified as glucuronic acid conjugates that demonstrate no documented severe toxicity [69]. These results confirm that the CE@BP-Gel hydrogel microspheres enable sustained release of bioactive components while exhibiting outstanding biocompatibility and hemocompatibility, positioning them as promising candidates for bone repair applications.

### 3.3 Immunomodulatory properties of hydrogel microspheres

**Fig. 5:**
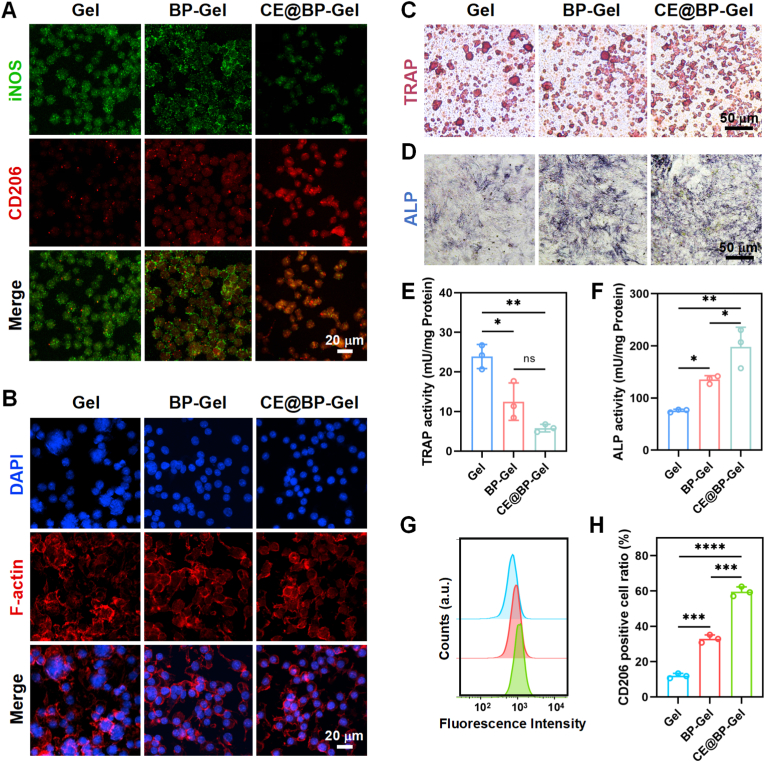
Immunomodulatory properties of the hydrogel microspheres. (A) Representative immunofluorescence images of iNOS and CD206 in RAW264.7 cells. (B) Morphology of preosteoclasts incubated with the hydrogel microsphere extracts. (C) TRAP staining of preosteoclasts cultured with the hydrogel microsphere extracts. (D) ALP staining of BMSCs co-cultured with RAW264.7 cells after 7 days of osteogenic induction. (E) TRAP activity assay in preosteoclasts after incubation with the hydrogel microsphere extracts. (F) ALP activity assay of BMSCs co-cultured with RAW264.7 cells after 7 days of osteogenic induction. (G) Immunomodulatory properties of the hydrogel microspheres based on flow cytometry analysis. (H) Quantification of CD206-positive RAW264.7 cells. n = 3, ∗p < 0.05, ∗∗p < 0.01, ∗∗∗p < 0.001 and ∗∗∗∗p < 0.0001.

In the repair of bone defects, immune regulation is crucially important. The repair of bone defects not only demands the differentiation of osteoblasts and bone formation but also requires reshaping the local microenvironment through immune regulation to suppress excessive inflammatory reactions [70,71]. In this study, RAW264.7 cells were treated with LPS to simulate the inflammatory response. As depicted in Fig. 5(A), after incubation with different hydrogel microsphere extracts in RAW264.7 cells, there were significant differences in polarization phenotypes. The RAW264.7 cells in the LPS-induced Gel group produced high levels of iNOS, while the expression level of CD206 was significantly lower. However, in the CE@BP-Gel group, RAW264.7 cells were significantly polarized towards the M2 type, accompanied by the inhibition of iNOS expression and a significant increase in the expression of CD206. The quantitative statistics indicated that the value of CD206/iNOS in the CE@BP-Gel group was significantly elevated compared to the Gel and BP-Gel groups (Fig. S8).

Osteoclasts are the key cells accountable for bone resorption during bone remodeling. The osteoclastic differentiation is regulated by multiple cytokines, among which RANKL serves as a crucial inducing factor [72]. RANKL promotes the differentiation and maturation of osteoclasts by binding to its receptor RANK and activating downstream signaling pathways. During the osteoclastic differentiation of RAW264.7 cells, the morphology gradually transforms from round mononuclear cells to multinucleated giant cells, ultimately forming mature osteoclasts with bone resorption functionality [73]. This process involves cytoskeleton reorganization, cell fusion, and the expression of specific markers, constituting an important model for investigating bone metabolic disorders.

In this work, RAW264.7 cells were induced to osteoclastic differentiation by 50 ng/mL RANKL, and the influence of hydrogel microspheres on osteoclast activity was assessed. As depicted in Fig. 5(B), significantly more multinucleated giant cells were observed in the Gel group, with a marked increase in cell volume and the emergence of apparent pseudopodia and protrusions at the cell margins. In contrast, the quantity of multinucleated giant cells was conspicuously reduced in the BP-Gel and CE@BP-Gel groups, and the cell nuclei were mostly independently distributed. Furthermore, mature osteoclasts stained with TRAP exhibited red cytoplasm and distinct nuclei, while negative staining cells appeared yellow. As shown in Fig. 5(C), the number of mature osteoclasts in the Gel group was significantly higher than in the BP-Gel and CE@BP-Gel groups. Both cell morphology and TRAP staining observations suggested that BP-Gel and CE@BP-Gel could inhibit RANKL-induced osteoclast differentiation of RAW264.7 cells.

On the other hand, the osteogenic activity of BMSCs co-cultured with RAW264.7 cells was evaluated. ALP staining in Fig. 5(D) indicated that the osteogenic differentiation of BMSCs was significantly enhanced in the CE@BP-Gel group. M2-type macrophages induced by CE@BP-Gel could induce the osteogenic differentiation of BMSCs through paracrine effects. Quantitative statistics of TRAP and ALP activity further demonstrated that the osteoclastic activity of RAW264.7 cells induced by CE@BP-Gel was suppressed, while the osteogenic performance was significantly elevated (Fig. 5(E, F)). Additionally, the proportion of CD206-positive M2-type markers in RAW264.7 cells was quantitatively evaluated by flow cytometry (Fig. 5(G, H)). The proportion of M2 polarization in RAW264.7 cells induced by CE@BP-Gel approximated 60 %, while in the Gel group and BP-Gel group, these values were 16 % and 35 %, respectively. Evidently, CE@BP-Gel can achieve immune-mediated bone regeneration by enhancing the M2 polarization of macrophages and inhibiting osteoclast differentiation.

### 3.4 Osteogenic and angiogenic properties evaluation

**Fig. 6:**
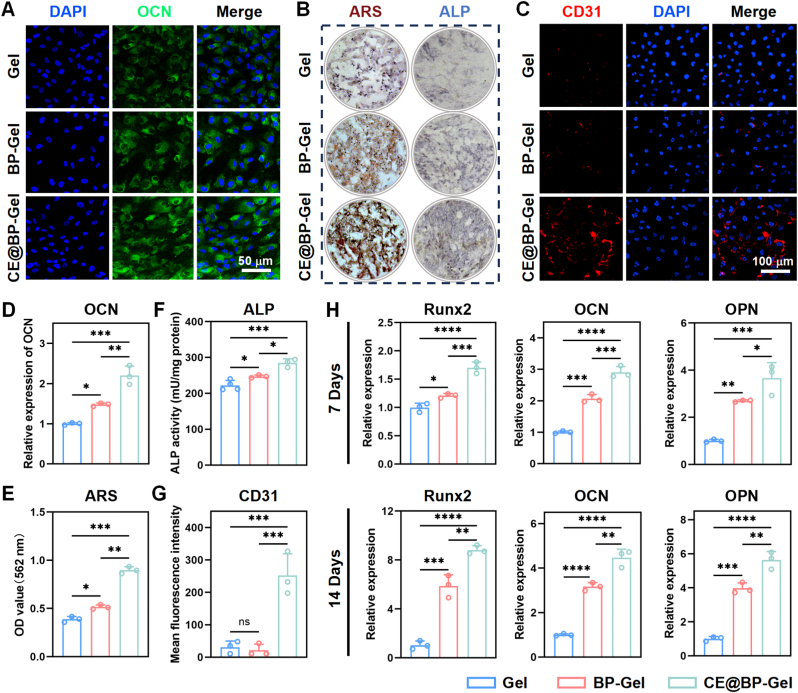
Osteogenic and angiogenic properties of the hydrogel microspheres. (A) Immunofluorescence images of OCN in BMSCs after 14 days of osteogenic induction. (B) ALP staining and ARS staining of BMSCs after 7 and 14 days of osteogenic induction. (C) Immunofluorescence images of CD31 in HUVECs. (D) Quantitative statistics of the mean fluorescence intensity (MFI) of OCN. (E) Quantitative assay of ARS staining solution. (F) ALP activity quantitative analysis on day 7. (G) Quantitative statistics of the MFI of CD31. (H) Expression of osteogenesis-related genes in BMSCs after osteogenic induction. n = 3, ∗p < 0.05, ∗∗p < 0.01, ∗∗∗p < 0.001 and ∗∗∗∗p < 0.0001.

It has been demonstrated that CE@BP-Gel can promote immune-mediated bone regeneration. The osteogenic differentiation of BMSCs directly mediated by CE@BP-Gel is presented in Fig. 6. Immunofluorescence staining revealed a significantly higher expression level of OCN in the CE@BP-Gel group compared to the Gel and BP-Gel groups (Fig. 6(A)). Quantitative analysis in Fig. 6(D) indicated that the expression of OCN was elevated in the BP-Gel group relative to the Gel group. Notably, after loading Cur@Exos, the expression of OCN was further significantly enhanced. OCN is one of the most abundant proteins in the bone matrix [74,75]. It performs multiple functions in bone metabolism, such as regulating bone mineralization, participating in bone remodeling, and modulating bone and glucose metabolism. The expression of OCN is commonly regarded as a marker of osteoblast maturation and is closely associated with bone formation activity [76]. The significant upregulation of OCN in the CE@BP-Gel group underscores its superior osteogenic activity.

During the osteogenic differentiation of BMSCs, the increase in ALP activity serves as another critical early marker. ALP hydrolyzes organic phosphates to generate inorganic phosphates, which is essential for the mineralization of the ECM [77]. As shown in Fig. 6(B–F), both ALP staining and ALP activity assays demonstrated that ALP expression in BMSCs within the CE@BP-Gel group was significantly elevated compared to the Gel and BP-Gel groups. The mineralization of the ECM involves the transformation of collagen fibers and non-collagenous proteins into bone tissue by osteoblasts [78]. This process begins with calcification points in the ECM, gradually forming hydroxyapatite crystals and ultimately forming mineralized bone tissue. ARS staining in Fig. 6(B) indicated that CE@BP-Gel accelerated calcium salt deposition in the ECM. The number of dark red calcified nodules in the CE@BP-Gel group was significantly higher than in the Gel and BP-Gel groups. The enhanced ECM mineralization further confirmed that CE@BP-Gel significantly promotes the osteogenic differentiation of BMSCs (Fig. 6(E)). Additionally, the expression levels of the osteogenesis-related genes (Runx2, OCN and OPN) in the CE@BP-Gel group were significantly higher than those in the Gel and BP-Gel groups, indicating the excellent osteogenic induction performance of CE@BP-Gel (Fig. 6(H)).

The aforementioned results indicate that CE@BP-Gel mediates bone regeneration via its potent immunomodulatory properties and directly induces the osteogenic differentiation of BMSCs. Previous studies have demonstrated that alendronate promotes osteogenic differentiation by modulating key signaling pathways in BMSCs, such as Wnt/β-catenin, BMP/Smad, and MAPK [[79], [80], [81]]. Additionally, alendronate enhances the expression of osteogenesis-related markers such as OCN and ALP in osteoblasts, thereby facilitating the synthesis and mineralization of the bone matrix [82]. On the other hand, both curcumin and the nucleic acid components of exosomes in Cur@Exos have been shown to possess significant osteogenic induction effects [83,84]. MSC-Exos contain diverse signaling molecules, such as miRNAs, mRNAs, and proteins, which can be internalized by BMSCs and modulate their osteogenic gene expression and cellular functions. Studies have demonstrated that miRNAs carried by MSC-Exos can target and activate signaling pathways associated with osteogenic differentiation in BMSCs [85].

Angiogenesis plays a critical role in bone formation by supplying essential oxygen and nutrients to the defect area, thereby supporting the growth and differentiation of bone cells [86]. The vascular network also facilitates the clearance of metabolic waste products such as carbon dioxide and lactic acid, maintaining tissue health. Additionally, vascular endothelial cells and other cells involved in the repair process, including osteoblasts and osteoclasts, migrate to the repair site via the vascular system [87]. Growth factors and cytokines (e.g., VEGF and FGF) released during angiogenesis significantly influence the proliferation and differentiation of osteoblasts. Therefore, effective angiogenesis is indispensable for the repair of bone defects. CD31 is an important transmembrane glycoprotein expressed on vascular endothelial cells that plays a crucial role in endothelial cell adhesion, migration, and signal transduction, particularly during angiogenesis [88]. As shown in Fig. 6(C), the expression of CD31 in HUVECs was significantly higher in the CE@BP-Gel group compared to the Gel and BP-Gel groups. Quantitative analysis of average fluorescence intensity confirmed that CD31 expression was markedly elevated in the CE@BP-Gel group, with no significant difference between the Gel and BP-Gel groups (Fig. 6(G)).

The migratory capacity of HUVECs was assessed using a wound healing assay (Fig. S9A). At 6 and 12 h, the CE@BP-Gel group exhibited significantly greater migration than the Gel and BP-Gel groups. Quantitative analysis further supported these findings, showing a higher migration rate in the CE@BP-Gel group, while no significant difference was observed between the Gel and BP-Gel groups (Fig. S9B). HUVECs were seeded onto Matrigel-coated cell plates to evaluate angiogenic potential and cultured under appropriate conditions. As illustrated in Fig. S10(A), HUVECs began to form tubular structures at 4 h, with tight junctions between the lumen and cells. At 6 h, it could be observed that CE@BP-Gel significantly induced the formation of a well-developed network structure, while microtubules were sparsely distributed in the Gel group and the BP-Gel group. Quantitative analysis revealed that the total vessel length in the CE@BP-Gel group was significantly longer than in the Gel and BP-Gel groups, with no significant difference between the latter two groups (Fig. S10B). The results from CD31 expression, cell migration, and tube formation assays indicate that CE@BP-Gel significantly promotes angiogenesis, while BP alone does not possess this capability.

### 3.5 Biomineralization and bone repair evaluation

**Fig. 7:**
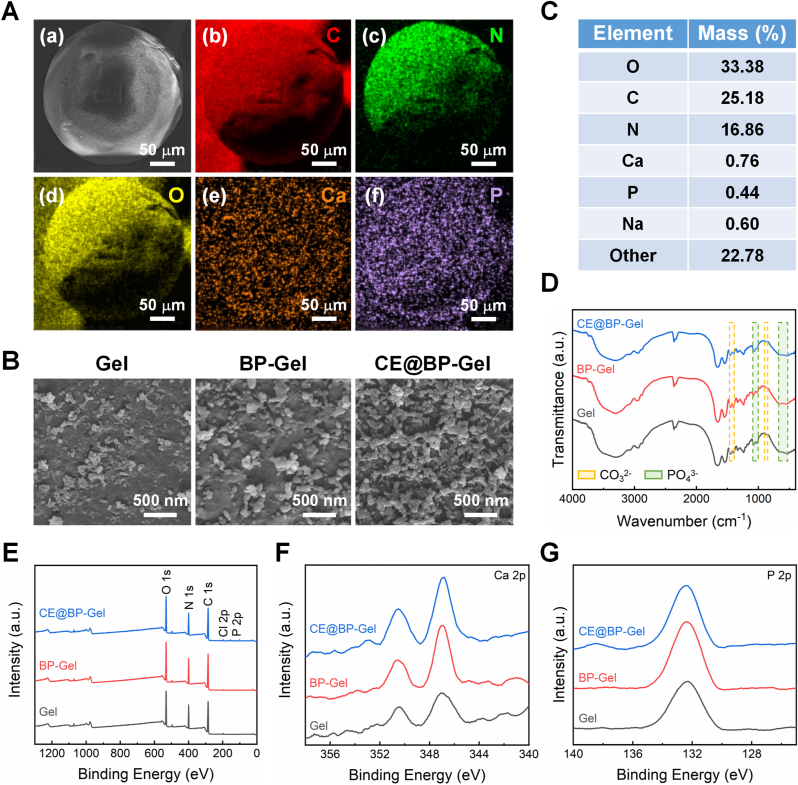
*In vitro* mineralization assessment of the hydrogel microspheres. (A) SEM and element distribution images of CE@BP-Gel after 14 days of mineralization in SBF solution. (B) SEM images of apatite nanoparticles on the surface of mineralized hydrogel microspheres. (C) Element mass percentages on the surface of CE@BP-Gel microspheres. (D) FTIR spectrum of the mineralized hydrogel microspheres. (E–G) XPS analysis of the mineralized hydrogel microspheres, (E) XPS survey spectra, (F) Ca 2p and (G) P 2p XPS spectra.

The biomineralization of biomaterials is a critical step in the bone repair process. Accelerating the deposition of surface hydroxyapatite enhances the integration of biomaterials with bone tissue, thereby improving the stability of implants [89]. Moreover, the mineralization process can release specific signaling molecules, such as calcium ions and phosphate ions, which activate signaling pathways within osteoblasts and promote osteogenic differentiation [90]. To evaluate the biomineralization capability of hydrogel microspheres, the microspheres were immersed in SBF at 37 °C for 14 days and subsequently freeze-dried. The surface deposition of apatite nanoparticles was examined using SEM. As shown in Fig. 7(A), a uniform distribution of C, N, O, Ca, and P elements was observed on the surface of the mineralized CE@BP-Gel microspheres. Notably, Ca and P elements were not detected on the surface of the non-mineralized CE@BP-Gel microspheres, indicating that these elements primarily originated from the surface-deposited apatite nanoparticles. The surface morphology of the mineralized hydrogel microspheres is illustrated in Fig. 7(B). Both BP-Gel and CE@BP-Gel microspheres showed significantly more nanoparticle deposits compared to the Gel group, which had fewer nanoparticles.

In biological systems, apatite predominantly exists as hydroxyapatite, with a chemical formula of Ca_5_(PO_4_)_3_OH. In ideal hydroxyapatite, the Ca/P ratio is approximately 1.67 [91]. However, various factors can cause deviations from this ideal ratio during biomineralization processes. As shown in Fig. 7(C), the elemental content on the surface of CE@BP-Gel exhibits a Ca/P ratio of 1.72, which closely approximates the theoretical value. The molecular structure of the mineralized hydrogel microspheres was characterized using FTIR spectroscopy, as depicted in Fig. 7(D). Characteristic absorption peaks at 560-600 cm^−1^ correspond to the bending vibration of PO_4_^3−^, while those at 1000-1100 cm^−1^ correspond to the symmetric stretching vibration of PO_4_^3−^. Additionally, vibration peaks attributed to CO_3_^2−^ were observed at 870-880 cm^−1^ and 1420-1450 cm^−1^. The presence of PO_4_^3−^ in the FTIR spectrum further confirms apatite formation.

Furthermore, XPS was employed to analyze the elemental composition on the surface of the mineralized hydrogel microspheres, as shown in Fig. 7(E–G). The Ca 2p and P 2p signals of CE@BP-Gel and BP-Gel were significantly stronger than those on Gel, providing further evidence that BP-modified hydrogels enhance apatite deposition. Due to the presence of two adjacent phosphate groups in the BP molecule, it exhibits high efficiency in coordinating with various metal ions (such as Ca^2+^ or Mg^2+^) [92]. BP can form stable complexes with Ca^2+^, leading to localized regions of high calcium concentration within the hydrogel, thereby promoting the nucleation and growth of apatite crystals.

**Fig. 8:**
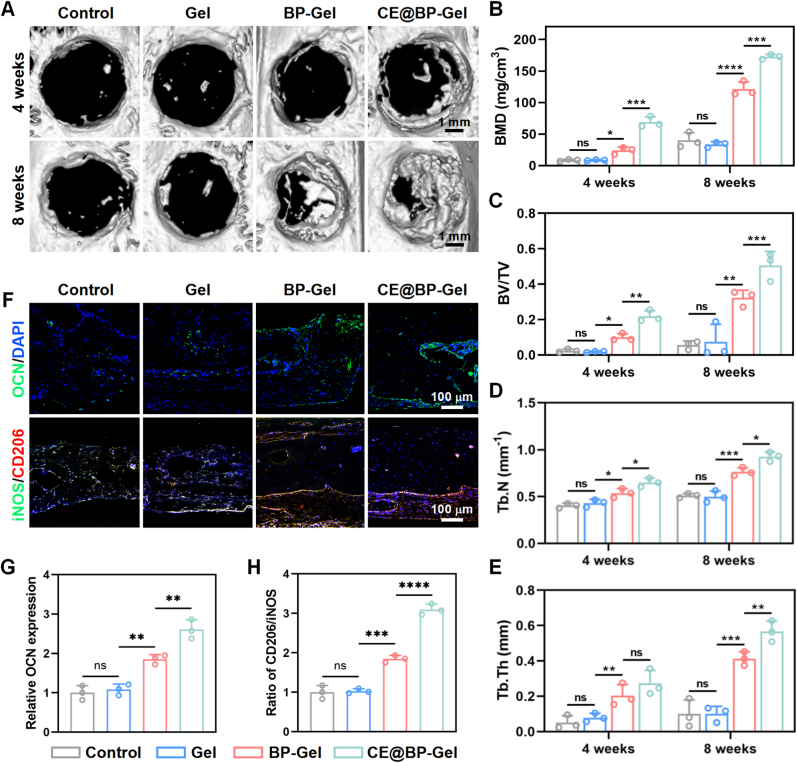
*In vivo* bone repair assessment of the hydrogel microspheres. (A) Micro-CT analysis of critical-sized cranial defects in SD rats. Quantitative statistics of (B) BMD, (C) BV/TV, (D) Tb.N and (E) Tb.Th in new bone tissue. (F) Immunofluorescence staining of OCN, iNOS and CD206 in the bone defect area. (G) Quantitative statistics of OCN in the bone defect area. (H) Quantitative statistics of CD206/iNOS in the bone defect area. n = 3, ∗p < 0.05, ∗∗p < 0.01, ∗∗∗p < 0.001 and ∗∗∗∗p < 0.0001.

The critical-sized cranial defect model was utilized to assess the *in vivo* osteogenic performance of the prepared composite hydrogel microspheres. Circular full-thickness defects with a 5-mm diameter were generated in the calvaria of male SD rats. The hydrogel microspheres were injected into the cranial defect, and bone regeneration was evaluated at 4 and 8 weeks after implantation (Fig. 8(A)). At the fourth week, although the bone defects in all groups were not completely repaired, a significantly increased new bone formation was observed in the CE@BP-Gel group. In the eighth week, the bone defects in the BP-Gel and CE@BP-Gel groups were almost completely filled with new bone tissue, while only limited new bone formation was present in the control group and the Gel group. Meanwhile, the quantitative statistical results based on Micro-CT also indicated the excellent performance of CE@BP-Gel in repairing critical-sized bone defects (Fig. 8(B-E)).

Immunofluorescence staining of the defect regions revealed markedly increased OCN expression intensity in the newly formed bone tissue of the BP-Gel and CE@BP-Gel groups relative to the Control and Gel groups (Fig. 8(F)). Conversely, the defects in the control and Gel groups were predominantly occupied by fibrous tissue, accompanied by fewer osteoblasts and reduced OCN expression levels (Fig. 8(G)). Additionally, immunofluorescence staining for macrophage markers indicated elevated iNOS expression in the fibrous tissue of the control and Gel groups, whereas the anti-inflammatory factor CD206 expression was significantly upregulated in the BP-Gel and CE@BP-Gel groups (Fig. 8(F)). Quantitative analysis of the CD206/iNOS ratio confirmed an elevated inflammatory status in the Control and Gel groups, while macrophages in the BP-Gel and CE@BP-Gel groups were polarized toward an anti-inflammatory phenotype favorable for bone regeneration (Fig. 8(H)). These *in vivo* bone formation results further validate the outstanding therapeutic efficacy of CE@BP-Gel in promoting bone defect repair.

### 3.6 Molecular mechanisms of immune regulation

Following bone tissue injury, the inflammatory response constitutes the initial phase of the repair process. While an appropriate inflammatory reaction aids in the removal of damaged tissue and pathogens, excessive inflammation may exacerbate tissue damage [93]. Ideal bone implant materials should possess active immunomodulatory properties, suppressing pro-inflammatory factors while promoting anti-inflammatory factor expression to maintain inflammatory balance and create favorable conditions for bone repair [94]. As a crucial component of the immune system, macrophages play multiple roles in tissue repair and regeneration, particularly exerting a key regulatory effect in the inflammatory phase. M1-type macrophages secrete pro-inflammatory cytokines and are involved in inflammatory reactions, while M2-type macrophages possess anti-inflammatory effects, regulating angiogenesis, fibroblast regeneration, and collagen production and facilitating tissue regeneration [95]. As previously described, CE@BP-Gel demonstrated excellent osteogenic, angiogenic, and immunomodulatory capabilities. Notably, in a co-culture system, CE@BP-Gel-induced M2-polarized RAW264.7 cells further promoted the osteogenic differentiation of BMSCs by mimicking the anti-inflammatory microenvironment during tissue repair and regeneration. Thus, elucidating the immunomodulatory mechanisms of CE@BP-Gel is critical to understanding its role in bone tissue repair.

**Fig. 9:**
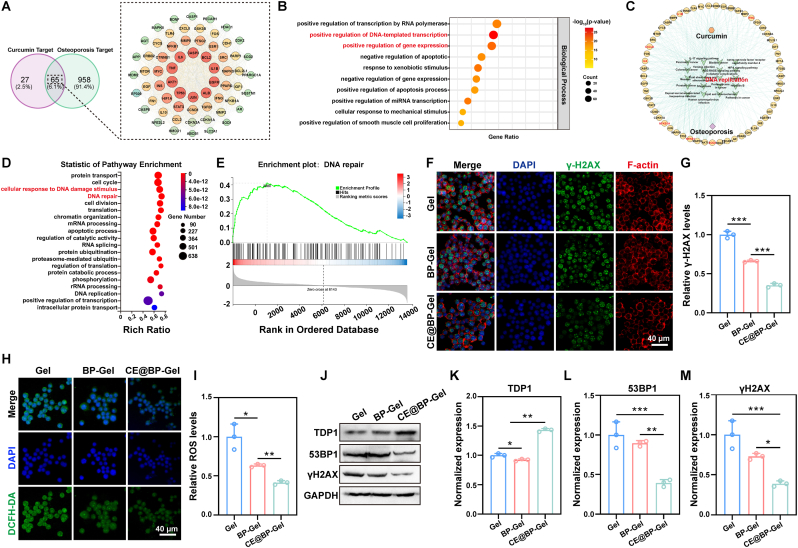
Immunomodulatory mechanisms of CE@BP-Gel. (A) Prediction of potential targets of curcumin in immune regulation. (B) Biological process of the potential targets. (C) Predicted drug-target-disease network of curcumin and immune regulation. (D) KEGG pathway enrichment analysis of differentially expressed genes based on RNA sequencing. (E) Gene set enrichment analysis (GSEA) of DNA repair-related gene sets. (F) Immunofluorescence staining of γ-H2AX in RAW264.7 cells. (G) Relative expression of γ-H2AX in RAW264.7 cells. (H) Expression of intracellular ROS in RAW264.7 cells. (I) Relative expression of intracellular ROS. (J) Expression of DNA repair-related proteins. (K–L) Quantitative statistics of protein expression. n = 3, ∗p < 0.05, ∗∗p < 0.01 and ∗∗∗p < 0.001.

Osteoimmunomodulation refers to the interaction between the immune and skeletal systems, which influences bone formation, repair, and disease states [96]. The immune system is intimately associated with tissue damage and regeneration. Hence, effectively regulating and manipulating immune responses to modulate the innate healing process is of paramount importance for successful bone repair [97]. Dysregulated osteoimmunomodulation is associated with various pathologies, such as osteoporosis, rheumatoid arthritis, and osteoarthritis. Successful bone repair requires equilibrium between bone formation and resorption, and excessive resorption may impede fracture healing [98]. Given the close relationship between bone repair and osteoporosis in terms of metabolic balance, this study selected osteoporosis as a disease model and employed network pharmacology to analyze the potential targets and molecular mechanisms of curcumin in bone defect treatment. Using the SwissTargetPrediction platform, CTD database, and SuperPred database, 92 potential targets of curcumin were predicted and consolidated. In parallel, 1023 osteoporosis and immunomodulation-related targets were identified via GeneCards and OMIM databases. As shown in Figs. 9(A), 65 overlapping targets were identified in the Venn diagram. These core targets were imported into the DAVID database for GO functional enrichment analysis and visualization. Fig. 9(B) displays the top 10 significantly enriched biological processes (BP), including positive regulation of gene expression, positive regulation of DNA-templated transcription, and response to xenobiotic stimulus. Key cellular components (CC) included protein-containing complex, nucleoplasm, and cytoplasm, while molecular functions (MF) such as enzyme binding, identical protein binding, and RNA polymerase II-specific DNA-binding transcription factor binding were prominent (Fig. S11). A drug-overlapping targets-pathways-disease network was constructed using Cytoscape, illustrating the multi-target and multi-pathway actions of curcumin in osteoporosis treatment (Fig. 9(C)).

Network pharmacology analysis revealed the potential therapeutic role of curcumin in osteoporosis. To further elucidate the immune-modulatory mechanisms of CE@BP-Gel, RNA sequencing was conducted. Compared with the Gel group, 296 genes were upregulated, and 431 genes were downregulated in RAW264.7 cells in the CE@BP-Gel group. KEGG pathway enrichment analysis of the differentially expressed genes (DEGs) identified the top 20 pathways, as shown in Fig. 9(D). The analysis indicated that cellular responses to DNA damage stimuli and damage repair play critical roles in the immune phenotypic transformation of RAW264.7 cells. Additionally, gene set enrichment analysis in Fig. 9(E) demonstrated positive enrichment of the DNA repair pathway. LPS can induce inflammatory responses in RAW264.7 cells, releasing various inflammatory factors such as TNF-α, IL-1β, and IL-6 [99]. These inflammatory factors activate intracellular signaling pathways, resulting in increased production of ROS and subsequent DNA damage. Curcumin has been reported to reduce intracellular ROS levels through its antioxidant properties, thereby inhibiting DNA damage [100]. Moreover, DNA damage repair is essential for maintaining genomic stability and normal cellular functions [101]. RNA sequencing results suggest that CE@BP-Gel may enhance DNA damage repair in RAW264.7 cells. However, the underlying mechanism remains to be elucidated.

γH2AX is the phosphorylated form of histone H2AX, which occurs at the Ser139 site in response to DNA double-strand breaks (DSBs) [102]. As a critical biomarker for DNA damage, γH2AX rapidly forms foci at the sites of DSBs, recruits repair proteins, and initiates the DNA damage response. As shown in Fig. 9(F), immunofluorescence staining was used to evaluate γH2AX expression in RAW264.7 cells. In all three groups, distinct γH2AX foci were observed in the nuclei, indicating that LPS-induced double-strand breaks had occurred and that the cells were actively engaged in DNA repair. Quantitative analysis in Fig. 9(G) revealed that the γH2AX expression level in the CE@BP-Gel group was significantly lower than in the Gel and BP-Gel groups, suggesting that CE@BP-Gel can mitigate LPS-induced DNA damage. Typically, LPS induces the production of inflammatory factors and oxidative stress in RAW264.7 cells, leading to increased intracellular ROS levels and subsequent DNA damage [103,104]. Fig. 9(H, I) demonstrates that LPS induced high levels of ROS in RAW264.7 cells, while CE@BP-Gel significantly reduced ROS production. The normal levels of ROS function as signaling molecules involved in cell signaling and physiological regulation. However, excessive ROS can cause DNA damage, including base oxidation, single-strand breaks, and double-strand breaks [105]. Therefore, the lower intracellular ROS levels observed in the CE@BP-Gel group likely contributed to the reduction in DNA damage.

When DNA damage occurs, γH2AX is rapidly phosphorylated at the Ser139 site near the damage site [106]. Subsequently, 53BP1 and other repair proteins are recruited to these sites, forming nuclear foci that facilitate chromosomal stability [107]. The TDP1 enzyme plays a critical role in repairing DNA single-strand breaks and certain types of double-strand breaks by removing covalently bound proteins from the ends of DNA strands, thereby eliminating obstacles in the DNA repair process [108]. The coordinated interaction and functional synergy among γH2AX, 53BP1, and TDP1 are essential for maintaining genomic stability [109]. As shown in Fig. 9(J), western blotting was used to evaluate the expression levels of these proteins. Quantitative analysis revealed that the expression levels of γH2AX and 53BP1 were significantly lower in the CE@BP-Gel group compared to the Gel and BP-Gel groups, while TDP1 expression was markedly increased (Fig. 9(K-M)). The reduced ROS levels in the CE@BP-Gel group corresponded to decreased γH2AX and 53BP1 expression, indicating less DNA damage. The upregulation of TDP1 suggests that cells are actively enhancing their DNA repair capacity in response to residual DNA damage. Therefore, it can be concluded that CE@BP-Gel effectively mitigates LPS-induced DNA damage and promotes DNA repair by upregulating TDP1 expression.

**Fig. 10:**
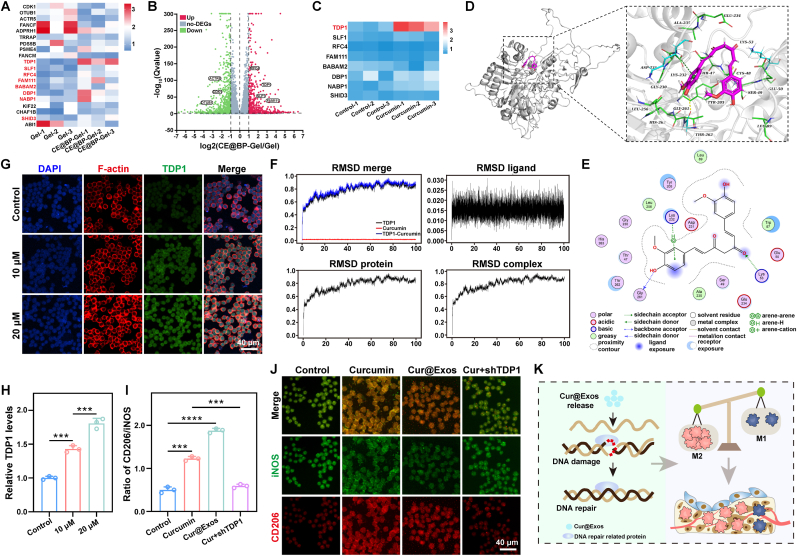
Molecular mechanism of CE@BP-Gel in DNA damage repair. (A) Gene expression heatmap of DNA damage repair. (B) Volcano plot of differentially expressed genes. (C) Expression of DNA damage repair-related genes in RAW264.7 cells induced by curcumin. (D) Molecular docking of curcumin with TDP1 target. (E) Local amplification of the binding site between curcumin and TDP1 protein. (F) Molecular dynamics simulation of curcumin/TDP1 complex. (G) Immunofluorescence staining of TDP1 in RAW264.7 cells treated with curcumin. (H) Relative expression of TDP1 in RAW264.7 cells. (I) Quantitative statistics of the ratio of CD206 to iNOS. (J) Immunofluorescence staining of CD206 and iNOS in RAW264.7 cells. (K) Schematic diagram of the immunomodulatory mechanism of the Cur@Exos. n = 3, ∗p < 0.05, ∗∗p < 0.01, ∗∗∗p < 0.001 and ∗∗∗∗p < 0.0001.

DNA damage repair is a critical process for maintaining the stability of genetic information in cells. Multiple DNA damage repair mechanisms exist within cells, and TDP1 plays a key role in single-strand break repair (SSBR) and base excision repair (BER) pathways [110]. In this study, it has been confirmed that CE@BP-Gel can accelerate DNA damage repair by upregulating TDP1. However, the specific molecular mechanism remains to be elucidated. Specifically, CE@BP-Gel contains multiple active components, including curcumin, nucleic acids, and proteins derived from exosomes. A precise understanding of the molecular mechanism underlying the effects of CE@BP-Gel on DNA damage repair will facilitate targeted regulation of these complex systems to meet more sophisticated immune modulation requirements. The differentially expressed genes associated with DNA damage repair, identified through RNA sequencing, were validated by qPCR and illustrated in Fig. 10(A). TDP1, SLF1, DPB1, and NABP1 gene expression levels were all elevated to varying degrees in the CE@BP-Gel group. This upregulation was also observed in the volcano plot of DEGs presented in Fig. 10(B). RAW264.7 cells were treated with 10 μM curcumin, and the expression levels of the aforementioned genes were evaluated. As shown in Fig. 10(C), the expression of TDP1 in the curcumin treatment group was significantly higher than in the control group, while the expressions of the other genes showed no significant differences. Therefore, it can be concluded that the curcumin carried by CE@BP-Gel significantly increased the expression of TDP1.

Molecular docking simulations revealed that curcumin can bind to the active pocket of TDP1 with low binding energy, as depicted in Fig. 10(D). Fig. 10(E) shows that curcumin can covalently bind to specific lysine and glycine residues on the TDP1 protein. Curcumin bound to the active pocket of TDP1 activates the catalytic activity of the enzyme, thereby enhancing its capacity to repair DNA damage. Binding free energy, a critical thermodynamic parameter characterizing the spontaneity of molecular interactions, serves as a key indicator for evaluating drug-target binding affinity. A negative binding free energy value signifies spontaneous molecular association, with lower values corresponding to stronger binding forces. It is generally accepted that binding free energy below −5 kcal/mol indicates favorable drug-target affinity [111]. In this investigation, the calculated binding free energy of −27.29 kcal/mol between curcumin and TDP1 protein demonstrates a robust and stable interaction between these molecular entities (Table S2). This quantitative result provides thermodynamic evidence for the strong binding capacity of curcumin to the TDP1 enzyme. The activated TDP1 can recognize and bind to the termini of damaged DNA, initiating the repair of DNA strand breaks. Additionally, molecular dynamics simulations demonstrated that the RMSD (Root Mean Square Deviation) of the TDP1/curcumin complex during the simulation was nearly identical to that of the TDP1 protein alone, indicating the high stability of the TDP1/curcumin complex (Fig. 10(F)). The backbone structure of TDP1 did not undergo significant conformational changes after docking with curcumin.

Immunofluorescence staining confirmed that the expression level of TDP1 in curcumin-treated RAW264.7 cells was significantly elevated (Fig. 10(G, H)). Moreover, as the concentration of curcumin increased from 10 μM to 20 μM, the expression of TDP1 further increased. To assess the role of TDP1 in immunomodulation, siRNA-mediated knockdown of TDP1 was performed in Fig. S12. Subsequent evaluation of M1/M2 macrophage polarization markers (CD206 and iNOS) revealed that curcumin or Cur@Exos treatment significantly increased the CD206/iNOS ratio compared to LPS-induced controls (Fig. 10(I, J)). In the control group, LPS induced RAW264.7 cells to produce high levels of iNOS, while CD206 expression was relatively weak. However, in RAW264.7 cells treated with curcumin or Cur@Exos, the ratio of CD206 to iNOS was significantly higher compared to the control group. However, in the TDP1-knockdown group (curcumin + shTDP1), CD206 expression was markedly reduced, underscoring the critical role of TDP1 in modulating macrophage polarization. Enhanced TDP1 expression in LPS-stimulated RAW264.7 cells may mitigate M1 polarization by repairing inflammation-induced DNA damage, thereby suppressing pro-inflammatory signaling. Additionally, TDP1 may influence macrophage polarization via transcriptional regulation or signaling pathway modulation. CE@BP-Gel facilitates DNA repair and promotes an M2-polarized immunomodulatory microenvironment via curcumin-mediated upregulation and activation of TDP1 (Fig. 10(K)).

Conventional engineering scaffolds often trigger acute foreign body reactions or persistent inflammation in bone defect repair, leading to impaired healing outcomes. The critical challenge in bone tissue engineering lies in effectively modulating the immune microenvironment to mitigate inflammatory responses while promoting osteogenesis [112]. Previous investigations have revealed that a timely transition from M1 to M2 macrophage polarization facilitates the resolution of proinflammatory reactions, thereby accelerating bone regeneration through coordinated vascularization, mesenchymal stem cell recruitment, osteogenic differentiation, and mineralization [113]. In this study, CE@BP-Gel-mediated curcumin delivery significantly upregulates TDP1 expression, which repairs ROS-induced DNA single-strand breaks under inflammatory conditions and preserves macrophage genomic integrity. This molecular mechanism effectively attenuates excessive activation of proinflammatory signaling pathways, particularly NF-κB. Reduced DNA damage accumulation suppresses M1 macrophage polarization while favoring M2 phenotype commitment, thereby remodeling the local immune microenvironment to support bone regeneration. The M2-polarized macrophages orchestrate tissue repair through dual mechanisms: secretion of anti-inflammatory cytokines (IL-10, TGF-β) to resolve excessive inflammation and production of pro-angiogenic factors (VEGF, HIF-1α) along with osteogenic mediators (BMP-2) to facilitate vascular neogenesis and osteoblast differentiation [[114], [115], [116]]. Furthermore, TDP1 activation diminishes intracellular ROS levels by downregulating γH2AX and 53BP1 expression, suppressing osteoclast differentiation markers (TRAP, NFATc1) and inhibiting inflammation-mediated bone resorption [117].

### 3.7 Innovation and translational potential

The poor solubility, structural instability, and low bioavailability of the small-molecule compound curcumin severely limit its application in treating bone-related diseases. A. Dahiya et al. physically adsorbed curcumin nanoparticles within the pore structure of 3D-printed calcium phosphate scaffolds, demonstrating enhanced osteogenic capacity, potent antitumor effects, and broad-spectrum antibacterial efficacy [118]. Y. Zheng et al. integrated curcumin-loaded calcium carbonate (CaCO_3_@Cur) nanoparticles into nanofiber implants to repair rat cranial defects [119]. While these strategies successfully loaded curcumin into composite scaffolds, they failed to address the inherent challenges of low solubility, poor bioavailability, and instability of free-form curcumin upon release. Recent studies suggest that nanocarrier-based delivery represents a more effective strategy to enhance curcumin bioavailability. N. Sarkar et al. encapsulated curcumin in liposomes embedded within 3D-printed calcium phosphate scaffolds with tailored porosity [120]. Over a two-month monitoring period, liposome-encapsulated curcumin exhibited superior controlled release compared to free curcumin. W. Li et al. reported that curcumin-loaded liposomes (Cur-Lip) alleviated senescence in BMSCs and restored their proliferative potential by activating mitophagy [121]. Although synthetic liposomes show promise for curcumin delivery, their long-term biosafety requires further validation. In contrast, MSC-Exos possesses inherent biocompatibility, avoiding immune rejection. Additionally, MSC-Exos carry specific membrane proteins that enable active targeting of bone defect sites through homologous cell recognition mechanisms, enhancing curcumin enrichment in pathological regions. By comparison, liposomes rely on passive diffusion or complex surface modifications for targeting, which may compromise their stability.

H. Lei et al. functionalized a silanized titanium alloy scaffold with human umbilical cord MSC-Exos, significantly enhancing osseointegration in an osteoporotic rat model [122]. The loaded exosomes rejuvenated senescent BMSCs, reduced senescence-associated secretory phenotype (SASP) factor secretion, and markedly improved BMSCs proliferation, migration, and osteogenic differentiation. However, exosomes chemically grafted onto scaffold surfaces are rapidly cleared by the immune system post-implantation, with no controlled release mechanism. Hydrogels provide an optimal platform for stable exosome loading and sustained release. X. Lu et al. developed an intelligent bilayer hydrogel co-loaded with diclofenac sodium and MSC-Exos, which effectively scavenged ROS, reprogrammed macrophages, attenuated post-traumatic inflammation, and promoted cartilage regeneration [123]. A hyaluronic acid/sodium alginate composite hydrogel with optimized porosity and biocompatibility enabled sustained MSC-Exos release, accelerating BMSC migration and differentiation for cartilage repair. S. Pan et al. encapsulated miR-29a-enriched MSC-Exos within injectable hydrogel microparticles (HMP@Agomir-29a-Exo) via microfluidic technology, achieving sustained exosome release in fracture microenvironments [124]. HMP@Agomir-29a-Exo significantly enhanced osteogenesis and angiogenesis by regulating the miR-29a/HDAC4/Runx2 axis, demonstrating robust bone regeneration and vascular repair capabilities both in vitro and *in vivo*. Building upon existing hydrogel-exosome therapeutic platforms, this study proposes a localized delivery strategy combining drug-loaded exosomes with functional hydrogels, broadening the applicability of hydrogel-exosome systems in specific clinical scenarios.

This study pioneers integrating MSC-Exos with curcumin via an endogenous drug-loading strategy to prepare Cur@Exos, which are further embedded into bisphosphonate-modified GelMA hydrogel microspheres. This composite system not only addresses the low bioavailability of curcumin resulting from its hydrophobicity but also sustains the activity of curcumin via the membrane structure of exosomes, thereby enabling efficient and targeted delivery. CE@BP-Gel microspheres locally inhibit osteoclast activity via bisphosphonate release, while Cur@Exos promotes macrophage polarization toward the anti-inflammatory M2 phenotype, reshaping the immune microenvironment in bone defect regions. Furthermore, curcumin enhances DNA damage repair by activating TDP1 enzyme activity, mitigating inflammation-driven oxidative stress accumulation, and revealing a novel molecular mechanism for bone repair. This dual-action mode, which integrates immunomodulation with direct osteogenesis and angiogenesis, surpasses the functional limitations of traditional bone repair materials. By integrating network pharmacology, molecular docking, and RNA sequencing, this study systematically elucidates the immunoregulatory network of CE@BP-Gel, with a particular emphasis on the critical role of TDP1-mediated DNA repair pathways in macrophage polarization. This establishes a novel technical framework for osteoimmunomodulation research.

CE@BP-Gel microspheres resolve critical challenges in bone repair, including immune dysregulation and insufficient osteogenic activity, through innovative material design and mechanistic exploration. This system demonstrates both scientific merit and clinical translational potential as a next-generation candidate for bone tissue engineering. In a rat critical-size cranial defect model, CE@BP-Gel microspheres exhibited remarkable bone regeneration, with immunofluorescence staining confirming their ability to upregulate anti-inflammatory factors and suppress proinflammatory cytokines, thereby improving the local immune microenvironment. This therapeutic system combines osteogenic, anti-inflammatory and angiogenic functions, effectively repairing complex bone defects, and demonstrates targeted therapeutic efficacy in patients with comorbidities such as chronic inflammation or osteoporosis. Microfluidic technology enables high-throughput, standardized production of hydrogel microspheres with strong scalability. The components (GelMA, bisphosphonates, curcumin) are industrially producible, and the injectable microspheres support minimally invasive surgery for irregular defect filling, ensuring high clinical adaptability. This strategy can be extended to other regenerative applications, such as cartilage repair or chronic wound healing. The exosome-based drug delivery platform is also adaptable to diverse therapeutics (small-molecule inhibitors or nucleic acids), offering the potential for treating inflammatory diseases or tumors via immunomodulation.

## 4 Conclusion

In this study, the fabricated CE@BP-Gel hydrogel microspheres have demonstrated promising application prospects in the repair of critical-sized bone defects. The CE@BP-Gel hydrogel microspheres exhibit excellent biocompatibility and can accelerate nano-apatite deposition, thereby effectively mimicking the microstructure of natural bone tissue. The sustained release of BP and Cur@Exos from CE@BP-Gel significantly suppresses the expression of inflammatory factors and inhibits osteoclast formation in RAW264.7 cells. Furthermore, CE@BP-Gel induces polarization of RAW264.7 cells towards the regenerative M2 phenotype by inhibiting ROS-induced DNA damage and upregulating the expression of the DNA damage repair enzyme TDP1. Apart from immune-mediated osteogenesis, CE@BP-Gel also directly promotes the osteogenic differentiation of BMSCs and the angiogenesis of HUVECs. Significantly enhanced bone regeneration and anti-inflammatory properties of CE@BP-Gel were observed in the cranial defects of rats. In summary, this study presents a novel approach for the in-situ treatment of critical-sized bone defects through the synergistic effects of immunomodulation, osteogenesis, and angiogenesis.

## CRediT authorship contribution statement

**Yunhui Si:** Writing – original draft, Methodology, Investigation, Formal analysis, Data curation, Conceptualization. **Shuao Dong:** Writing – original draft, Methodology, Investigation. **Mengsha Li:** Methodology, Formal analysis. **Jiaying Gu:** Methodology, Formal analysis. **Manxuan Luo:** Methodology, Formal analysis. **Xiaohan Wang:** Methodology. **Zhiwei Wang:** Methodology. **Xiaorong Li:** Writing – review & editing, Supervision. **Chao Zhang:** Writing – review & editing, Supervision, Funding acquisition.

## Declaration of competing interest

The authors declare that they have no known competing financial interests or personal relationships that could have appeared to influence the work reported in this paper.

## Data Availability

Data will be made available on request.
