# Supplementary material for: Curcumin-encapsulated exosomes in bisphosphonate-modified hydrogel microspheres promote bone repair through macrophage polarization and DNA damage mitigation
Source: Mater Today Bio. 2025 May 15;32:101874. doi: 10.1016/j.mtbio.2025.101874 (PMC12145554; doi:10.1016/j.mtbio.2025.101874)
Supplement: Multimedia component 1 [file mmc1.docx]

**Supplementary Information**

**Curcumin-Encapsulated Exosomes in Bisphosphonate-Modified Hydrogel Microspheres Promote Bone Repair through**

**Macrophage Polarization and DNA Damage Mitigation**

Yunhui Si**^a,1^**, Shuao Dong**^a,1^**, Mengsha Li**^c,1^**, Jiaying Gu**^a^**, Manxuan Luo**^b^**,

Xiaohan Wang**^b^**, Zhiwei Wang**^d^**, Xiaorong Li**^b,*^**, Chao Zhang**^a,*^**

**^a^** School of Biomedical Engineering, Shenzhen Campus of Sun Yat-sen University, Shenzhen, Guangdong, 518107, PR China

**^b^** The Seventh Affiliated Hospital of Sun Yat-sen University, Shenzhen, Guangdong, 518107, PR China

**^c^** School of Materials Science and Engineering, Sun Yat-sen University, Guangzhou, Guangdong, 510275, PR China

**^d^** Southern Marine Science and Engineering Guangdong Laboratory (Zhuhai), Zhuhai, Guangdong, 519000, PR China

**^1^** These authors contributed equally to this work.

**^*^** Corresponding author.

1. **mail addresses:**

lixr235@mail.sysu.edu.cn (X. Li)

zhchao9@mail.sysu.edu.cn (C. Zhang)

1. **Experimental Methods**

**1.1. Synthesis of GelMA and BP-GelMA**

GelMA was synthesized by dissolving 5 g of gelatin in 50 mL of PBS under continuous stirring at 50℃ until complete dissolution. Subsequently, 3 mL of methacrylic anhydride was added to the gelatin solution, and the reaction mixture was stirred at 50℃ for 3 h. The solution was centrifuged at 8000 rpm for 10 min to remove unreacted components, and the supernatant was collected. The solution was diluted with a twofold volume of preheated ultrapure water to terminate the reaction. The product was dialyzed (molecular weight cut-off: 14 kDa) in ultrapure water at 37℃ for 7 days, followed by freeze-drying to obtain a white foamy GelMA powder stored at -20℃ in the dark.

For bisphosphonate-functionalized GelMA (BP-GelMA), 0.1305 g of alendronate trihydrate was dissolved in 5 mL of ultrapure water to prepare a 0.08 M solution. Next, 5 mL of 50% glutaraldehyde was added, and the mixture was reacted at 45℃ overnight to synthesize aldehyde-modified BP (BP-CHO). After standing for 20 minutes, BP-CHO was washed with cold acetone. Subsequently, 60 mg of BP-CHO was mixed with 10 mL of 10% (w/v) GelMA aqueous solution and incubated in a 37℃ water bath overnight. The final product was dialyzed for 3 days, lyophilized to yield yellow porous BP-GelMA, and stored at -20℃.

**1.2. Cell Culture**

Primary BMSCs were isolated from the femoral and tibial bone marrow of male SPF-grade SD rats. The SD rats were subjected to euthanasia by inhaling an excessive amount of CO_2_. Then, the femurs and tibias were isolated and soaked in 75% ethanol for sterilization. The femur and tibia were incised on both sides to expose the bone marrow cavity. Subsequently, the bone marrow cavity was flushed with a culture medium containing serum, and the bone marrow cells were rinsed into a centrifuge tube. The rinsed bone marrow cell suspension was centrifuged at 2000 rpm for 10 minutes. The supernatant was removed, and the cell pellet was resuspended with the culture medium. The cell suspension was introduced into the culture dish and subsequently incubated in a cell culture incubator. Following a 24-hour incubation period, the medium was replaced to remove non-adherent cells while retaining the adherent BMSCs. Subsequently, the culture medium was changed every two days, and the BMSCs were passaged when they reached 80% confluence. BMSCs were cultured in a complete medium consisting of 89% DMEM/F12 basal medium (Gibco, USA), 10% fetal bovine serum (FBS, ExCell Bio, China), and 1% penicillin-streptomycin (P/S, Gibco, USA). This study used third- or fourth-generation BMSCs for *in vitro* experiments. RAW264.7 cells were cultured in a complete medium composed of 89% DMEM basal medium (Gibco, USA), 10% FBS, and 1% P/S. Passage was carried out when the cells grew to 80% confluence. HUVECs were cultivated in a complete medium formulated with 89% DMEM basal medium, 10% FBS, and 1% P/S. All cells were cultured in a humidified incubator containing 5% CO_2_ at 37℃.

**1.3. Characterization of exosomes**

The morphology of exosomes was observed by a 120 kV transmission electron microscopy (TEM, JEM1400, JEOL, Japan). Briefly, the exosome suspension was dropped onto a 300-mesh copper grid, fixed with 2.5% glutaraldehyde, and negatively stained with 1% phosphotungstic acid. TEM observation was performed after natural drying. The specific markers on the surface of exosomes, such as TSG101, CD63, and HSP70, are detected through western blotting. The concentration and particle size distribution of exosomes were determined by nanoparticle tracking analysis (NTA, ZetaView, Particle Metrix, Germany).

**1.4. Exosome labeling and uptake assay**

Exosomes were labeled with PKH67 (Beijing Fluorescence Biotechnology) according to the manufacturer’s protocol. Unbound dye was removed by ultracentrifugation (100,000 g, 1 h). BMSCs, HUVECs, and RAW264.7 cells were incubated with PKH67-labeled exosomes for 12 h. Cells were fixed with 4% paraformaldehyde, stained with FITC-phalloidin (cytoskeleton) and DAPI (nucleus), and imaged via confocal laser scanning microscopy (CLSM, Leica Stellaris, Germany).

**1.5. Exosome release profiling**

Exosome release was quantified using a bicinchoninic acid (BCA) assay. Hydrogel microspheres (50 mg/mL in PBS) were incubated at 37℃. At predetermined intervals, 50 μL of supernatant was sampled and replaced with fresh PBS. Exosome concentration was determined against a standard curve.

**1.6. Characterization of the hydrogel microspheres**

The surface morphology and elemental distribution of the freeze-dried hydrogel microspheres were observed using a scanning electron microscope (SEM, Gemini 500, Zeiss, Germany) equipped with energy dispersive spectroscopy (EDS, UltimMax100, Oxford, UK). The chemical structure of the hydrogel microspheres was characterized using Fourier transform infrared spectroscopy (FTIR, IRAffinity-1S, Shimadzu, Japan). The freeze-dried hydrogel microspheres were mixed with potassium bromide (KBr) and pressed into a tablet, and the transmission spectrum was collected in the mid-infrared region (4000-400 cm^-1^). The surface chemical state of the hydrogel microspheres was characterized by X-ray photoelectron spectroscopy (XPS, Nexsa G2 X, Thermo Scientific, USA). In order to evaluate the mechanical properties of the composite hydrogels, cylindrical hydrogel specimens with a diameter of 8 mm and a thickness of 3 mm were subjected to compression tests on a material testing machine (68SC-2, Instron, USA) at a rate of 1 mm/min. The compression modulus was calculated from the slope of the linear portion of the compression curve at 10% strain. MCR302e rotational rheometer (Anton Paar, Austria) was used to measure the rheological properties of the composite hydrogels. For the purpose of evaluating the dynamic stability of the hydrogels, the fluctuations in the storage modulus (G’) and loss modulus (G”) of cylindrical hydrogel specimens (with a diameter of 8 mm and a height of 3 mm) were monitored at varying oscillation frequencies (0.1 Hz to 10 Hz) under a constant temperature of 37℃ and a strain of 0.5%. In addition, time mode scans were performed at 37℃ with an applied strain of 1% and a constant frequency of 1 Hz. In order to evaluate the degradation behavior, 50 mg of freeze-dried hydrogel microspheres were added to 1 mL of PBS solution containing 0.5 U/mL type II collagenase and incubated in a water bath oscillator at 37℃. The hydrogel microspheres were collected, freeze-dried, and weighed at a specified time.

**1.7. Sterilization procedure for hydrogel microspheres**

Implementing CE@BP-Gel in practical applications necessitates sterile production protocols and low-temperature gamma irradiation to control microbial contamination. Critical procedures, including exosome-hydrogel prepolymer mixing, are performed under stringent aseptic conditions within laminar flow hoods to prevent microbial ingress. Sterility assurance is further achieved through 0.22 μm membrane filtration of hydrogel prepolymer solutions, a well-established method for sterilizing exosome suspensions and gel matrices. For terminal sterilization of final CE@BP-Gel products, low-dose gamma irradiation (25 kGy, compliant with ISO 11137-2 standards) is employed. This optimized sterilization protocol effectively eliminates microbial contaminants while maintaining exosome bioactivity and hydrogel mechanical integrity.

**1.8. Biocompatibility of the hydrogel microspheres**

The biocompatibility of the hydrogel microspheres was evaluated through hemolysis testing, cytotoxicity assays, and live/dead staining. Fresh blood collected from the tail vein of SD rats was mixed with an anticoagulant ACD solution (Zeye Biotechnology) to prevent clotting. Plasma and erythrocytes were separated via centrifugation at 1,000 ×g for 10 min. The erythrocyte pellet was washed three times with PBS to remove residual plasma. For hemolysis testing, 10 mg of hydrogel microspheres were incubated with 1 mL of diluted erythrocyte suspension (4% v/v in PBS) at 37℃ for 1 h. After incubation, the supernatant was centrifuged at 1,000 ×g for 5 min, and 100 μL of supernatant was transferred to a 96-well plate. Absorbance at 540 nm was measured using a microplate reader. PBS and 0.5% Triton X-100 served as negative and positive controls, respectively.

For cytotoxicity assessment, BMSCs (passage 3) were seeded in 96-well plates at a density of 2 × 10^4^ cells/mL (100 μL/well) and cultured for 24 h. The medium was replaced with hydrogel microsphere extracts, and cells were cultured for 1, 3, and 5 days. At each time, 10% CCK-8 reagent (Beyotime, China) was added, followed by incubation for 2 h at 37℃. Absorbance at 450 nm was measured to calculate cell viability. Live/dead staining was performed using Calcein-AM (2 μM, EFL, China) and propidium iodide (1.5 μM, EFL, China). After 1 and 3 days of incubation with hydrogel extracts, BMSCs were stained and imaged via CLSM.

**1.9. Intracellular ROS assay**

The ROS level in RAW264.7 cells was evaluated by DCFH-DA staining. RAW264.7 cells were seeded in 24-well plates at 1×10^4^ cells/well density and stimulated with 100 ng/mL LPS for 24 hours to simulate an inflammatory environment. Subsequently, the culture medium was substituted with the extract of hydrogel microspheres, and the cells were further cultured for 3 days. The old culture medium was discarded, and the cells were washed once with a serum-free culture medium. 10 μM of DCFH-DA dye solution (MedChemExpress, USA) was added to each well and incubated in the dark at 37℃ and 5% CO_2_ for 30 minutes. Next, the cells were washed twice with serum-free medium to remove the unbound dye. The expression level of intracellular ROS was observed using CLSM, and the average fluorescence intensity was quantified using Image J (NIH, USA).

**1.10. Osteogenesis-related gene expression**

After 7 and 14 days of osteogenic induction, BMSCs were harvested, and the total RNA was extracted using an RNA extraction kit (Sparkjade, China). The concentration and purity of RNA were measured using a NanoDrop Microvolume Spectrophotometer (ThermoFisher, USA). Subsequently, RNA was reverse-transcribed to complementary DNA (cDNA) using the PrimeScript RT kit (TaKaRa, Japan). The expression levels of osteogenesis-related genes were quantitatively analyzed by means of the CFX96 Real-Time Detection system (Bio-Rad, USA) and SYBR Premix Ex Taq II (TaKaRa, Japan). The primer sequences of the target genes are presented in Table S1. The relative expression levels of osteogenesis-related genes were calculated by the 2^-ΔΔCt^ method, and GAPDH was employed as the housekeeping gene for standardization.

**1.11. Evaluation of the migratory capacity of HUVECs**

The scratch healing assay was used to evaluate the migration ability of HUVECs. HUVECs were inoculated into 6-well plates at a density of 1×10^5^ cells/well and cultured until a monolayer of cells was formed. The medium was discarded and a 10 μL pipette tip was used to slide vertically along the bottom of the well plate to create a uniform scratch on the monolayer of cells. The cells were gently washed with the culture medium to remove the cell debris generated during the scratching process, and the initial scratch profile was recorded. Subsequently, a serum-free medium containing extracts of hydrogel microspheres was added for continued culturing for 6 hours and 12 hours. The healing of the scratch area was observed using an inverted microscope, and the cell migration rate was quantitatively calculated using Image J (NIH, USA).

**1.12. Tube formation assay**

The matrigel stored at -20℃ was thawed on ice. The melted Matrigel (Corning, USA) was added to pre-cooled 96-well plates at a volume of 50 μL/well using a pre-cooled pipette and incubated in a 37℃ incubator for 30 min. The digested HUVECs were resuspended in a culture medium containing hydrogel microsphere extract and seeded on the solidified matrigel at a density of 1.5×10^4^ cells/100 μL. The culture plate seeded with cells was placed in a cell culture incubator and cultured at 37℃ and 5% CO_2_. The morphological changes and the formation of tubular structures of HUVECs were observed through a microscope. Image J software was used to quantify parameters such as the length of tubular structures and the number of branching points.

**1.13. Western blotting**

RAW264.7 cells were inoculated in 6-well plates, and the cells were collected when the confluence was between 80-90%. The cells were gently washed twice with pre-cooled PBS to remove residual serum proteins. 1 mL of lysis buffer containing protease inhibitors, phosphatase inhibitors and phenylmethylsulfonyl fluoride was added to each well for cell lysis. The lysate was mixed with SDS-PAGE loading buffer at a ratio of 1:4 and boiled in boiling water for 10 min to denature the protein. The protein samples were loaded onto lanes of polyacrylamide gels for SDS-PAGE separation at room temperature. Then, the proteins were transferred to a PVDF membrane (ThermoFisher, USA). The PVDF membrane was blocked with 5% skimmed milk for 2 hours at room temperature and then incubated with primary antibody at 4℃ overnight. After the PVDF membrane was washed three times with TBST (Sigma-Aldrich, Germany), it was incubated with the HRP-conjugated secondary antibody at room temperature for 2 hours. The protein bands were detected with Super ECL Plus chemiluminescent reagent (Epson, China), and the intensities of the bands were quantitatively analyzed by using Image J.

**1.14. Biomineralization assay**

Microspheres (5 mg) were immersed in 1.5× simulated body fluid (SBF, Yuanye Bio-Technology) at 37℃ for 14 days, with SBF refreshed every 48 h. Mineral deposition was analyzed via SEM, FTIR, and XPS.

**1.15. RNA sequencing**

RAW264.7 cells were inoculated into 6-well plates at a density of 1 × 10^5^ per well and induced by adding 100 ng/mL of LPS for 24 hours. Subsequently, the culture medium was replaced with one containing the extract of hydrogel microspheres, and the cells were cultured for 3 days. RNA was extracted by employing a Trizol reagent (Invitrogen, USA). The Beijing Genomics Institute (China) carried out whole-genome sequencing on the NovaSeq 6000 platform (Illumina, USA), generating approximately 20 million 150-bp paired-end reads for each sample. Low-quality reads and adapter sequences were removed using Trimmomatic, and quality control was conducted with FASTQC. Differential gene expression analysis was performed by employing the DESeq2 R package, and KEGG pathway enrichment analysis was conducted using KOBAS software to clarify gene functions. Differentially expressed genes were identified based on a p-value threshold of less than 0.05, and q-values were calculated by applying multiple comparison corrections, with the significance level set at q-values less than 0.05.

**1.16. Molecular dynamics simulation**

The molecular dynamics simulations were conducted using the Gromacs 2022.3 version software in this research. For the pretreatment of small molecules, the AmberTools22 software package was adopted in this study. The small molecules were parameterized with the GAFF force field, and the Gaussian 16W software was utilized to carry out hydrogenation treatment and calculate the RESP electrostatic potential for the small molecules. The calculated potential data were integrated into the topological file of the molecular dynamics system. The simulation process was performed under a constant temperature of 310 K and a standard atmospheric pressure (1 Bar), employing the Amber99sb-ILDN force field. The Tip3p water model was selected as the solvent, and an appropriate amount of Na^+^ was added to neutralize the total charge of the entire simulation system. The molecular dynamics simulation system was initially subjected to energy minimization via the steepest descent method, followed by a 100,000-step isothermal-isochoric ensemble (NVT) and isothermal-isobaric ensemble (NPT) equilibrium simulations. The coupling constant was set at 0.1 ps, and the duration for each was 100 ps. Eventually, a free molecular dynamics simulation was executed with 5,000,000 steps, a step size of 2 fs, and the total simulation duration reached 100 ns. After the simulation was completed, the trajectory data were processed using the software’s embedded analysis tools, and the root mean square deviation (RMSD) of the movement trajectories of each amino acid was calculated.

**Table S1.** Primer sequences used for qPCR of BMSCs

| Gene |  | Primer sequence (5' to 3') |
| --- | --- | --- |
| Runx2 | Forward | CAGTATGAGAGTAGGTGTCCCGC |
|  | Reverse | AAGAGGGGTAAGACTGGTCATAGG |
| OCN | Forward | CCCCCTCTAGCCTAGGACC |
|  | Reverse | ACCAGGTAATGCCAGTTTGC |
| OPN | Forward | ACAGCATCGTCGGGACCAGACTCGT |
|  | Reverse | GGTAGTGAGTTTTCCTTGGTCGGCG |
| GADPH | Forward | CCTTCCGTGTTCCTACCC |
|  | Reverse | CAACCTGGTCCTCAGTGTAG |

**Table S2. Curcumin/TDP1 binding free energy calculation results**

| Energy Component | Annotation | Average (kcal/mol) | SD (Prop.) |
| --- | --- | --- | --- |
| ΔE_VDWAALS_ | van der Waals energy | -42.21 | 1.74 |
| ΔE_EL_ | Electrostatic energy | -17.4 | 2.88 |
| ΔE_GB_ | Polar solvation energy | 38.1 | 1.96 |
| ΔE_Surf_ | Non-polar solvation energy | -5.78 | 0.04 |
| ΔG_Gas_ | Molecular mechanics energy | -59.61 | 3.36 |
| ΔG_Solv_ | Solvation energy | 32.32 | 1.96 |
| Δ_Total_ | Total Energy | -27.29 | 3.89 |

The MMGBSA analysis of TDP1-curcumin binding revealed a total binding free energy (Δ_Total_) of -27.29 ± 3.89 kcal/mol, demonstrating substantial binding capacity between the two entities. Van der Waals interactions (ΔE_VDWAALS_ = -42.21 ± 1.74 kcal/mol) and electrostatic contributions (ΔE_EL_ = -17.40 ± 2.88 kcal/mol) were identified as the primary driving forces, reflecting hydrophobic complementarity and charge interactions, respectively. Solvation effects (ΔG_Solv_ = 32.32 ± 1.96 kcal/mol) exhibited partial antagonistic effects on the binding process, with polar solvation energy (ΔE_GB_ = 38.10 ± 1.96 kcal/mol) counteracting electrostatic interactions while nonpolar solvation energy (ΔE_Surf_ = -5.78 ± 0.04 kcal/mol) provided stabilizing contributions to hydrophobic regions. Despite these solvation-mediated energy penalties, the combined van der Waals and electrostatic stabilization (-59.61 kcal/mol) substantially outweighed the desolvation energy cost, resulting in an overall favorable binding free energy. This thermodynamic profile confirms the persistent binding propensity between TDP1 and curcumin in aqueous environments, supported by the net negative Δ_Total_ value.


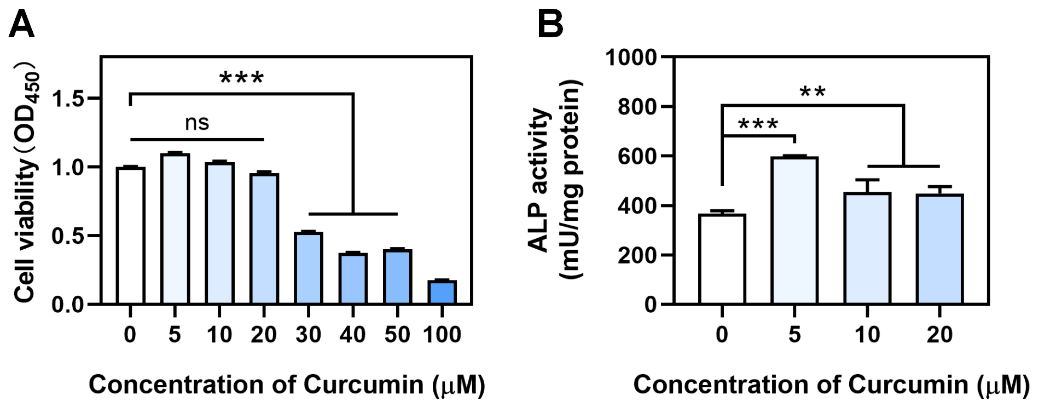


**Fig. S1. Effects of the curcumin concentration on the biological activity of BMSCs. (A) Evaluation of the proliferation activity of BMSCs based on CCK-8 assay. (B) ALP activity of BMSCs after 7 days of osteogenic induction.** n = 3, *p < 0.05, **p < 0.01 and ***p < 0.001.


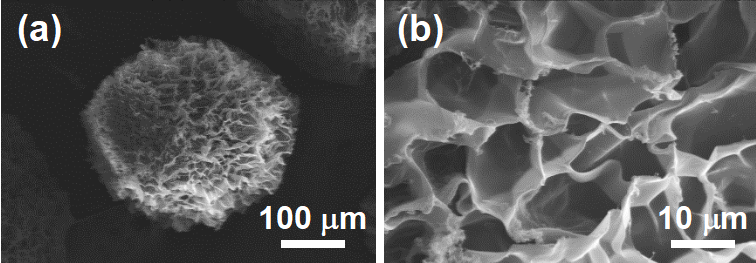


**Fig. S2. SEM images of the CE@BP-Gel microspheres**


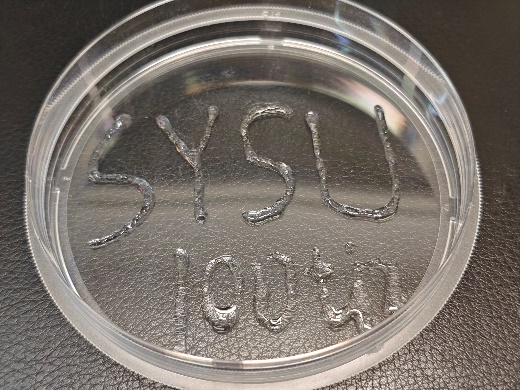


**Fig. S3. Visualization of the injectable CE@BP-Gel microspheres.**





**Fig. S4. Compressive stress-strain curves of CE@BP-Gel after 14 days of mineralization.**


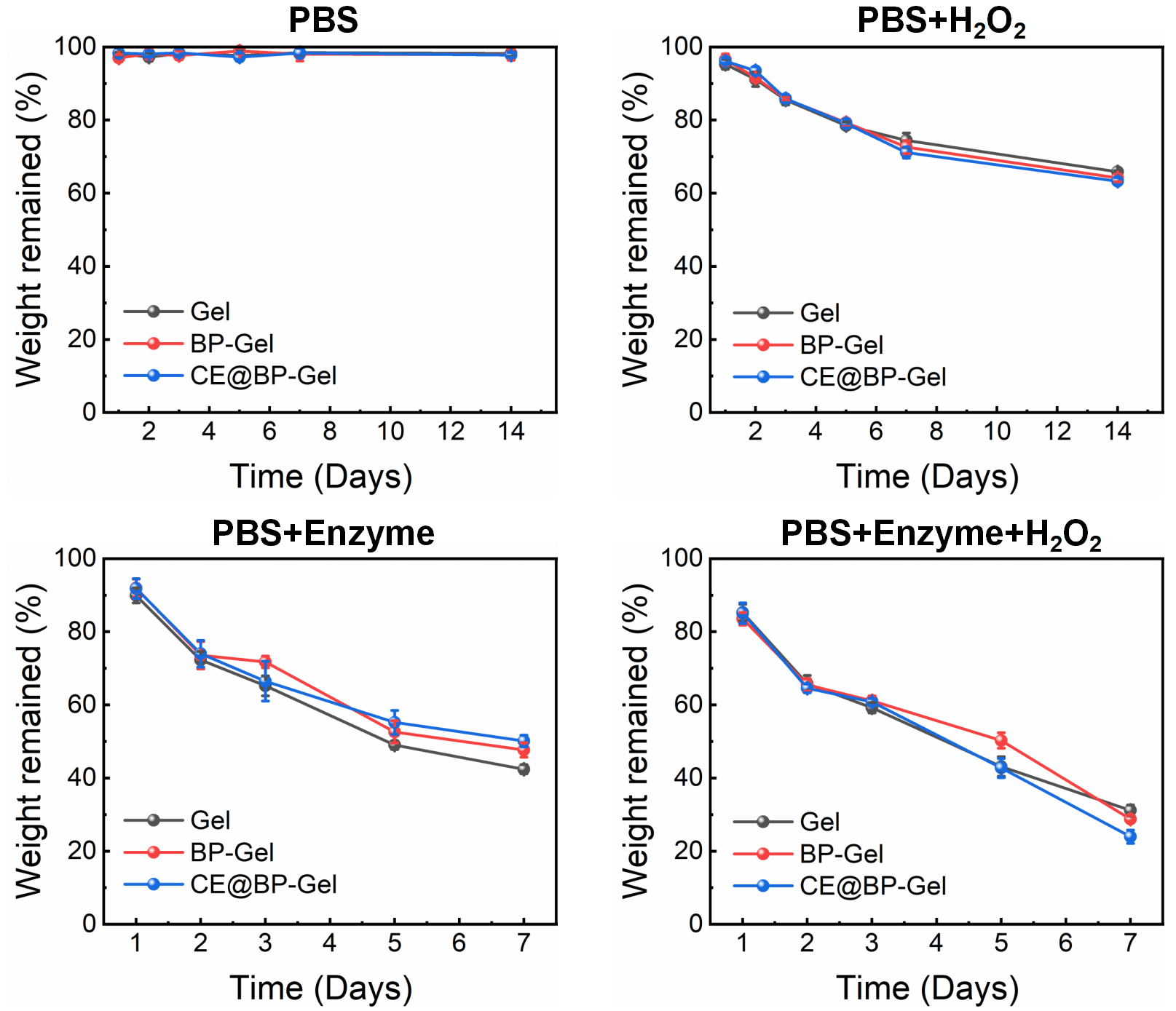


**Fig. S5. Degradation behavior of the hydrogel microspheres in PBS containing 0.5 U/mL type II collagenase and 100 mM H_2_O_2_.**


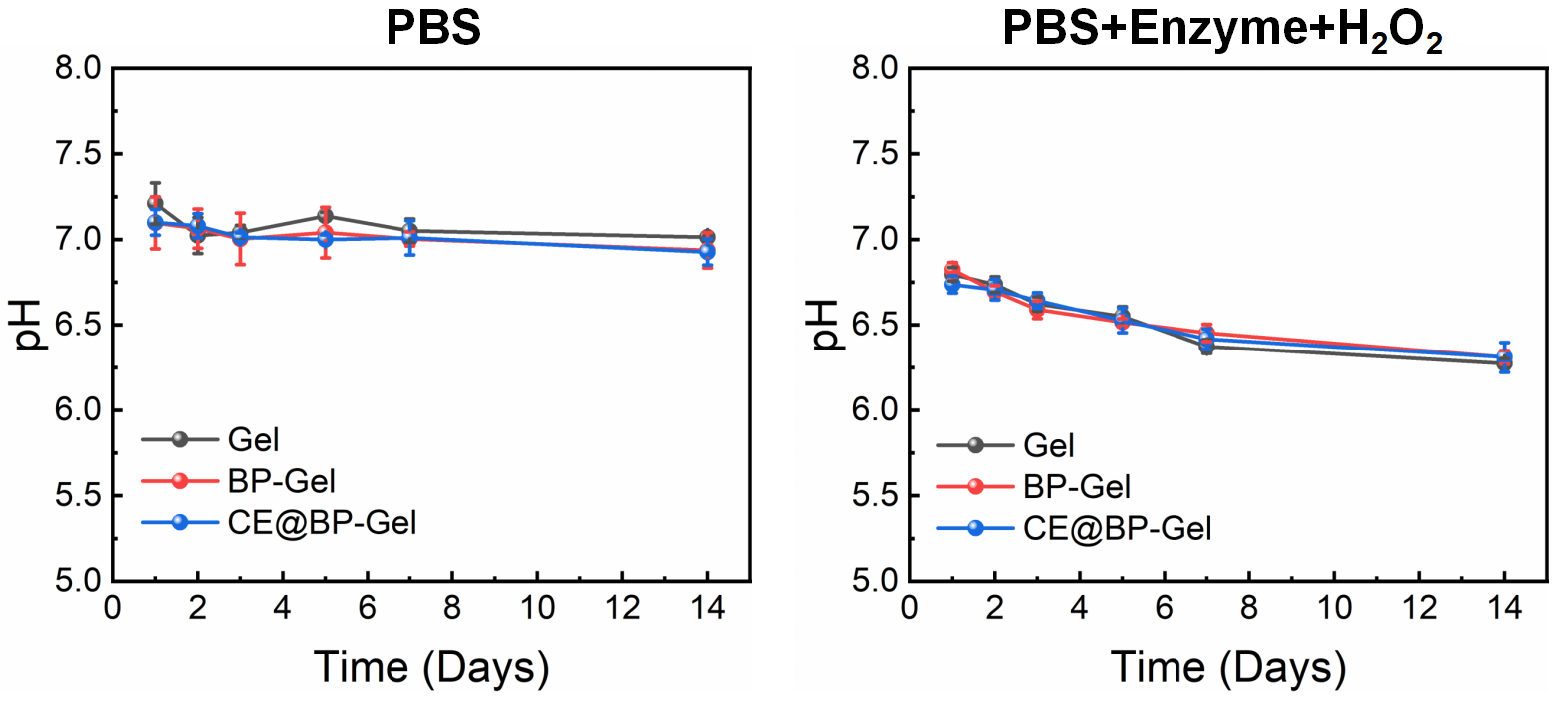


**Fig. S6. The variation of pH value during the degradation process of hydrogels.**





**Fig. S7. Assessment of the swelling behavior of the composite hydrogels.**


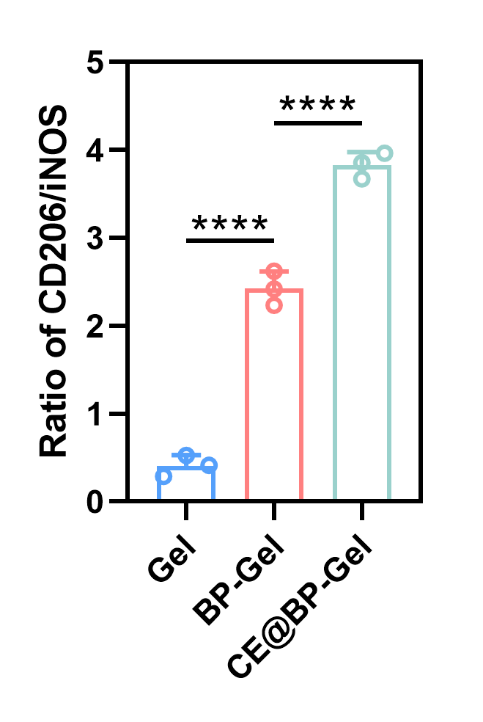


**Fig. S8. Quantitative statistics of the ratio of CD206 and iNOS in RAW264.7 cells.** n = 3, *p < 0.05, **p < 0.01, ***p < 0.001 and ****p < 0.0001.


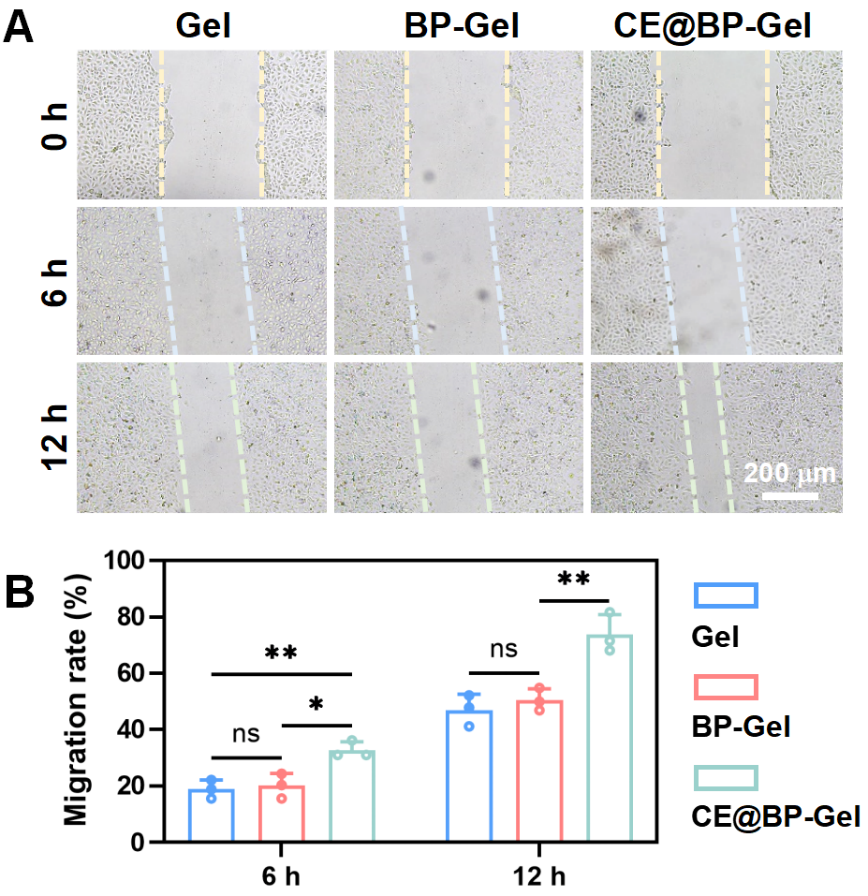


**Fig. S9. (A) Wound healing assay on cell migration of HUVECs. (B) Quantitative statistics of cell migration rate.** n = 3, *p < 0.05 and **p < 0.01.


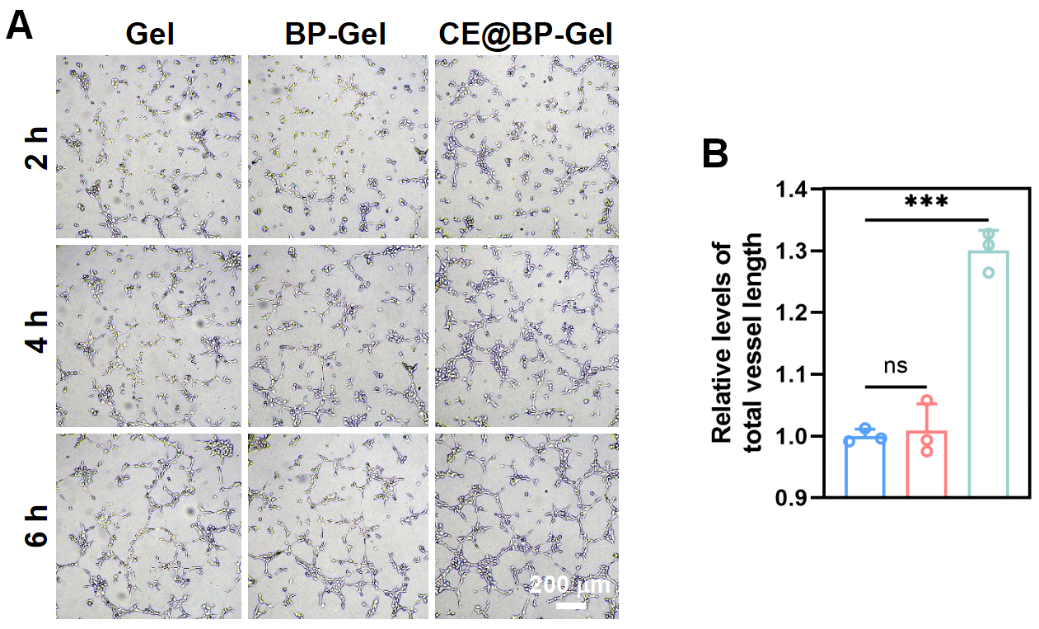


**Fig. S10. (A) The microscopic images of HUVECs tubular structure formation at different time points. (B) Quantitative statistical analysis of the total vessel length.** n = 3, *p < 0.05, **p < 0.01 and ***p < 0.001.


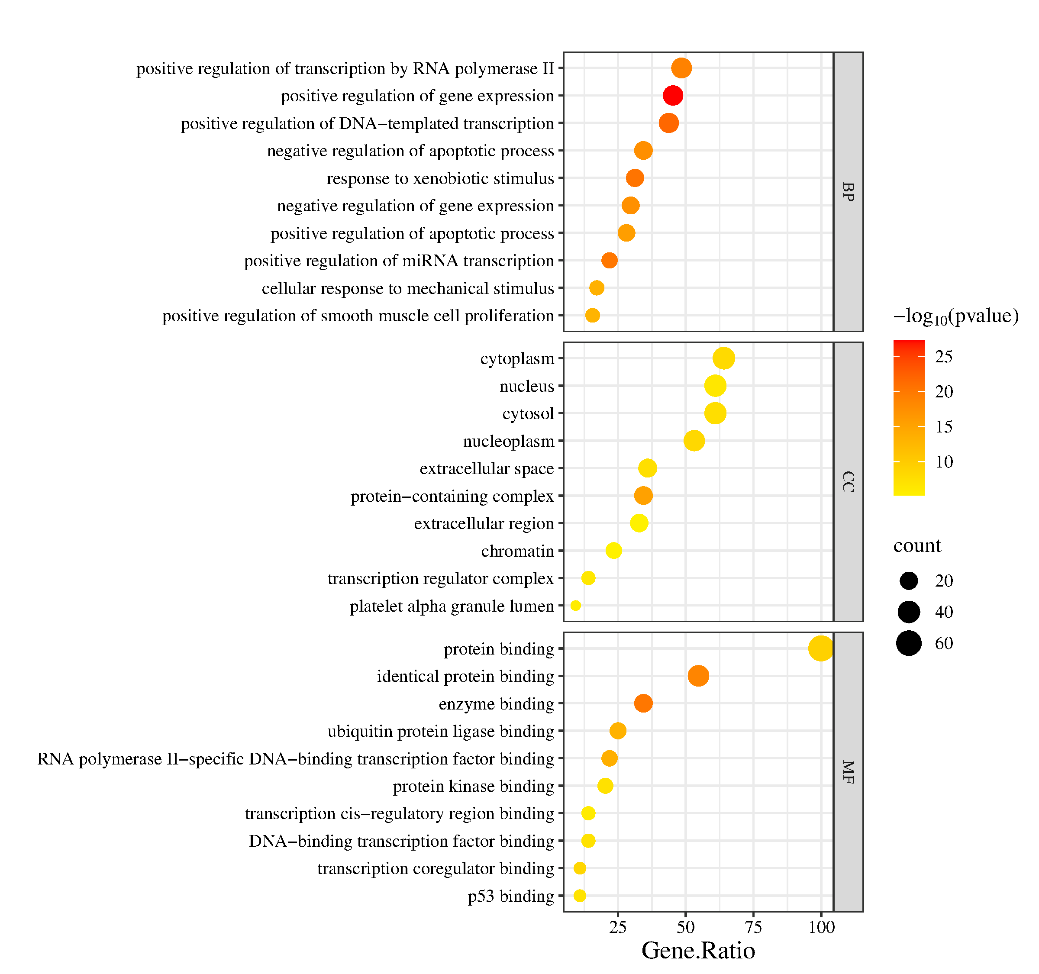


**Fig. S11. Bubble chart of GO function enrichment results.**


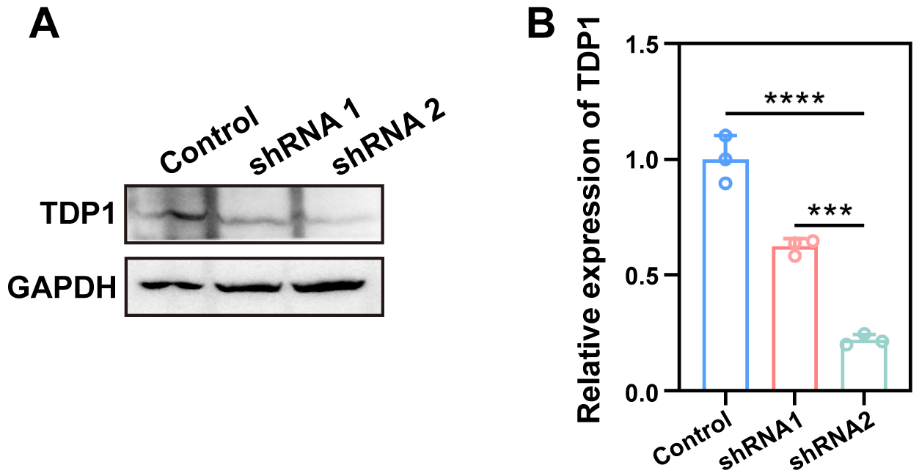


**Fig. S12. Validation of TDP1 knockdown by western blotting. (A) Western blot analysis of TDP1 gene knockdown. (B) Quantitative statistics of TDP1 protein expression.**
